# Supplementary material for: Health technology assessment of diagnostic tests: a state of the art review of methods guidance from international organizations
Source: Int J Technol Assess Health Care. 2023 Feb 21;39(1):e14. doi: 10.1017/S0266462323000065 (PMC7614237; doi:10.1017/S0266462323000065)
Supplement: Supplementary file 1 [file S0266462323000065sup001.zip › S0266462323000065sup001.docx]

**Supplementary file 1: Stage 1 data extraction form**

| **HTA Organisation** | **Reviewer / Date** |
| --- | --- |
|  |  |
| **Which documents did you rely on to compile this summary of the HTA organisation’s process for evaluating tests?** *e.g. Methods Guide, Process Guide, Scoping Methods Document* | |
|  | |
| **Pre–evidence review processes** | |
| **Where do the suggestions for which tests *(topics)* to review come from (referral source)?** *e.g. industry, research recommendations, clinicians, policy makers* | |
|  | |
| **Are there any entry criteria?** *e.g. CE mark for NICE DAP* | |
|  | |
| **How is the scope developed from the referral?** | |
|  | |
| **What is the focus of the scope?** *e.g. MSAC – focus is on detailed understanding of PICO, role of new test and mapping out care pathways* | |
|  | |
| **Evidence Review Methods and Process** | |
| **Does the process involve collection of evidence on the test(s)?** | |
|  | |
| **Who performs the evidence collection for the clinical effectiveness review (and if applicable cost-effectiveness review/analysis) and how is this funded?** *Designed to capture applicant–led reviews (e.g. NICE MTEP) vs reviews done by independent review centres (e.g. PenTAG or Warwick Evidence for NICE DAP)* | |
|  | |
| **Excluding issues of cost-effectiveness, what evidence should be collected?** | |
|  | |
| **How should the evidence be searched for?** | |
|  | |
| **How should the quality of evidence be assessed? And how should quality be incorporated into conclusions?** *e.g. QUADAS-2* | |
|  | |
| **How should the evidence be summarised and synthesised?** | |
|  | |
| **What evidence hierarchy/framework is used (if any) to judge the relative strength and importance of the evidence?** *e.g. GRADE* | |
|  | |
| **Is cost-effectiveness assessed?** *Referring to methods for any type of cost analysis (e.g. cost–utility, ‘cost–effectiveness’, cost–minimisation, budget impact analysis)* | |
|  | |
| **How should cost-effectiveness be assessed?** e*.g. systematic review ± de–novo modelling; recommendation to use specific analytic approaches, as listed above. Is the selection of utility measures considered an important part of the process? e.g. by providing guidance on how utility measures should be selected?* | |
|  | |
| **Where health economic modelling is used, how should the evidence collected be incorporated into that model?** *Also referred to as ‘linked evidence modelling/approach/methods’, what evidence should be linked with what? How is linked evidence defined? Also, how are the clinical effectiveness review and cost–effectiveness study linked together – e.g. are clin effectiveness review results used to inform the HE model?* | |
|  | |
| **Tail: Post–Evidence Review Processes** | |
| **What approach is used to develop the evidence (and information on cost-effectiveness if applicable) into guidance?** *e.g. NICE DAP and MSAC use a committee to consider all consultations as well as evidence reviews* | |
|  | |
| **How is guidance disseminated?** | |
|  | |
| **Top–Tail: Whole Process** | |
| **Are test developers involved in any part of the process?** | |
|  | |
| **How are test developers involved in the process?** *i.e. scope, collation of evidence, interpretation of evidence review, production of final guidance* | |
|  | |
| **Are the public involved in any part of the process?** | |
|  | |
| **How are the public involved in the process?** *(as above)* | |
|  | |
| **Are experts involved in the process?** | |
|  | |
| **How are experts involved in the process?** *(as above)* | |
|  | |
| **Specify location of process diagram [Enter URL if this is online only].** *i.e. flow diagram showing process steps.* | |
|  | |
| **Highlight any other important information in document (e.g. page refs/figs)** | |
|  | |
| **Key fields to extract for all other organisations** *any fields that stand out as being key to producing most rigorous HTA of tests* | |
|  | |
| **Other – anything else interesting on process or methods** | |
|  | |
