# Supplementary material for: Health technology assessment of diagnostic tests: a state of the art review of methods guidance from international organizations
Source: Int J Technol Assess Health Care. 2023 Feb 21;39(1):e14. doi: 10.1017/S0266462323000065 (PMC7614237; doi:10.1017/S0266462323000065)
Supplement: Supplementary file 1 [file S0266462323000065sup001.zip › S0266462323000065sup002.docx]

**Supplementary file 2: Summary of responses from HTA organisations**

Contents

[Agency for Healthcare Research and Quality (AHRQ, USA) 4](#_Toc96343077)

[Sources used for information 4](#_Toc96343078)

[HTA setting 4](#_Toc96343079)

[Processes for identifying diagnostic topics for HTA reports 4](#_Toc96343080)

[Process for scoping HTA diagnostic topics 5](#_Toc96343081)

[Process for involving stakeholders 6](#_Toc96343082)

[Processes for developing evidence into guidance and disseminating guidance 7](#_Toc96343083)

[Overview of guidance on methods for Health Technology Assessments of tests 7](#_Toc96343084)

[Methods for reviewing clinical evidence of diagnostic tests 7](#_Toc96343085)

[How should quality be assessed? 7](#_Toc96343086)

[How should evidence be synthesised? 8](#_Toc96343087)

[How should the strength of the body of evidence be determined? 9](#_Toc96343088)

[Methods for conducting health economics assessments 10](#_Toc96343089)

[Special features noted 10](#_Toc96343090)

[Questions to AHRQ 10](#_Toc96343091)

[Canadian Agency for Drugs and Technologies in Health (CADTH) 12](#_Toc96343092)

[Sources used for information 12](#_Toc96343093)

[HTA setting 12](#_Toc96343094)

[Processes for identifying diagnostic topics for HTA reports 13](#_Toc96343095)

[Process for scoping HTA diagnostic topics 13](#_Toc96343096)

[Process for involving stakeholders 14](#_Toc96343097)

[Processes for developing evidence into guidance and disseminating guidance 14](#_Toc96343098)

[Overview of guidance on methods for Health Technology Assessments of tests 14](#_Toc96343099)

[Methods for reviewing clinical evidence of diagnostic tests 15](#_Toc96343100)

[General approach 15](#_Toc96343101)

[Detailed methods for evaluating the cost-effectiveness of diagnostic tests 16](#_Toc96343102)

[Special features noted 16](#_Toc96343103)

[Questions to CADTH 17](#_Toc96343104)

[Institute for Quality and Efficiency in Health Care (Institut für Qualität und Wirtschaftlichkeit im Gesundheitswesen (IQWiG, Germany) 19](#_Toc96343105)

[Sources used for information 19](#_Toc96343106)

[HTA setting 19](#_Toc96343107)

[Processes for identifying diagnostic topics for HTA reports 20](#_Toc96343108)

[Process for scoping HTA diagnostic topics 20](#_Toc96343109)

[Process for involving stakeholders 20](#_Toc96343110)

[Processes for developing evidence into guidance and disseminating guidance 21](#_Toc96343111)

[Overview of guidance on methods for Health Technology Assessments of tests 21](#_Toc96343112)

[Methods for reviewing clinical evidence of diagnostic tests 21](#_Toc96343113)

[Special features noted 23](#_Toc96343114)

[Questions to IQWiG 23](#_Toc96343115)

[Medical Services Advisory Committee (MSAC, Australia) 25](#_Toc96343116)

[Sources used for information 25](#_Toc96343117)

[HTA setting 25](#_Toc96343118)

[Processes for identifying diagnostic topics for HTA reports 26](#_Toc96343119)

[Process for scoping HTA diagnostic topics 27](#_Toc96343120)

[Process for involving stakeholders 30](#_Toc96343121)

[Processes for developing evidence into guidance and disseminating guidance: 30](#_Toc96343122)

[Overview of guidance on methods for Health Technology Assessments of tests 31](#_Toc96343123)

[Methods for reviewing clinical evidence of diagnostic tests 31](#_Toc96343124)

[What evidence should be collected? 32](#_Toc96343125)

[Searching for evidence 33](#_Toc96343126)

[How should quality be assessed? 34](#_Toc96343127)

[How should ***evidence*** be synthesised? 34](#_Toc96343128)

[How should the strength of the body of evidence be determined? 37](#_Toc96343129)

[Overall interpretation of evidence for tests 38](#_Toc96343130)

[Methods for conducting health economics assessments 39](#_Toc96343131)

[Special features noted of the MSAC methods and processes 41](#_Toc96343132)

[Questions to MSAC 42](#_Toc96343133)

[National Institute for Health and Care Excellence Diagnostics Assessment Programme (NICE DAP, England and Wales) 46](#_Toc96343134)

[Sources used for information 46](#_Toc96343135)

[HTA setting 46](#_Toc96343136)

[Processes for identifying diagnostic topics for HTA reports 46](#_Toc96343137)

[Process for scoping HTA diagnostic topics 47](#_Toc96343138)

[Process for involving stakeholders 48](#_Toc96343139)

[Processes for developing evidence into guidance and disseminating guidance 49](#_Toc96343140)

[Overview of guidance on methods for Health Technology Assessments of tests 50](#_Toc96343141)

[Methods for reviewing clinical evidence of diagnostic tests 50](#_Toc96343142)

[Methods for conducting health economics assessments 51](#_Toc96343143)

[Special features noted 52](#_Toc96343144)

[Questions to NICE 52](#_Toc96343145)

[Swedish Agency for Health Technology Assessment and Assessment of Social Services (SBU, Sweden) 53](#_Toc96343146)

[Sources used for information 53](#_Toc96343147)

[HTA setting 53](#_Toc96343148)

[Processes for identifying diagnostic topics for HTA reports 53](#_Toc96343149)

[Process for scoping HTA diagnostic topics: 53](#_Toc96343150)

[Process for involving stakeholders 54](#_Toc96343151)

[Processes for developing evidence into guidance and disseminating guidance 54](#_Toc96343152)

[Overview of guidance on methods for Health Technology Assessments of tests 54](#_Toc96343153)

[Methods for reviewing clinical evidence of diagnostic tests: 55](#_Toc96343154)

[Methods for conducting health economics assessments 56](#_Toc96343155)

[Special features noted 56](#_Toc96343156)

[Questions to SBU 56](#_Toc96343157)

[National Health Care Institute (Zorginstituut Nederland) (ZIN, Netherlands) 58](#_Toc96343158)

[Sources used for information 58](#_Toc96343159)

[HTA setting 58](#_Toc96343160)

[Processes for identifying diagnostic topics for HTA reports 58](#_Toc96343161)

[Process for scoping HTA diagnostic topics 59](#_Toc96343162)

[Process for involving stakeholders 59](#_Toc96343163)

[Processes for developing evidence into guidance and disseminating guidance 60](#_Toc96343164)

[Overview of guidance on methods for Health Technology Assessments of tests 60](#_Toc96343165)

[Methods for reviewing clinical evidence of diagnostic tests 60](#_Toc96343166)

[Special features noted 62](#_Toc96343167)

[Questions to ZIN 62](#_Toc96343168)

## Agency for Healthcare Research and Quality (AHRQ, USA)

Responses from Christine Chang and Craig Umscheid, on behalf of AHRQ

### Sources used for information

1. ***Agency for Healthcare Research and Quality: A Profile.*** Content last reviewed March 2018. Agency for Healthcare Research and Quality, Rockville, MD. <https://www.ahrq.gov/cpi/about/profile/index.html> [last accessed 10^th^ June 2021]
2. ***EHC Program Topic Nomination and Selection***. EHC AHRQ web page, available from: <https://effectivehealthcare.ahrq.gov/about/epc/nomination/> [last accessed 20^th^ July 2020].
3. ***Evidence–based Practice Centers***. EHC AHRQ web page, available from: <https://effectivehealthcare.ahrq.gov/about/epc> [last accessed 24^th^ July 2020].
4. ***About the Evidence Synthesis Process.*** EHC AHRQ web page, available from: <https://effectivehealthcare.ahrq.gov/about/epc/evidence-synthesis> [last accessed 24^th^ July 2020].
5. ***Evidence-based Practice Center Editorial Review Process.*** EHC AHRQ web page, available from: <https://effectivehealthcare.ahrq.gov/about/epc/review> [last accessed 24^th^ July 2020].
6. ***Evidence-based Practice Center Evidence Report Surveillance Process.*** EHC AHRQ web page, available from: <https://effectivehealthcare.ahrq.gov/about/epc/update> [last accessed 24^th^ July 2020].
7. ***Methods Guide for Medical Test Reviews.*** AHRQ Publication No. 12-EC017. Rockville, MD: Agency for Healthcare Research and Quality; June 2012. Downloaded 20^th^ July 2020.
8. ***EPC Methods Guide for Comparative Effectiveness Reviews***. Available at <https://effectivehealthcare.ahrq.gov/products/cer-methods-guide> [last accessed 6^th^ June 2021].

### HTA setting

Health technology assessment (HTA) in the United States of America (US) is conducted by several organisations, however in 1999 the Agency for Healthcare Research and Quality (AHRQ) was established as the lead Federal agency for improving the safety and quality of the US’s health care system, including HTA which falls within the remit of AHRQ’s Effective Health Care Program (EHC) [1]. AHRQ refer to ‘HTAs’ as ‘comparative effectiveness reviews’ [8], which are conceived as systematic reviews of the evidence on tests, possibly augmented by modelling. They are not located in a system for making decisions, and so do not include separate guidance documents or formal recommendations. However AHRQ has a partnership with US Centers for Medicare and Medicaid Services (CMS) where AHRQ provides a technology assessment to inform CMS coverage decisions.

1. Pre–evidence review processes for HTAs of tests: we looked for information on:

- *Where suggestions for which tests to review come from (referral source)*
- *Whether there are any entry criteria (e.g. regulatory conformity such as CE mark)*
- *Whether a formal scoping phase is recommended or mandated, and if so how the scope should be developed from the referred topic, and what the scope’s focus should be.*

### Processes for identifying diagnostic topics for HTA reports

Proposals for any HTA topics can come from any source, including healthcare professionals, researchers, payers and patients [2]. Topic prioritisation processes are reported for any topic, with no special considerations for diagnostic topics [2,8].

“*A topic prioritization group representing stakeholder and scientific perspectives evaluates topic nominations*” [8: ‘Identifying, Selecting and Refining Topics for Comparative Effectiveness Systematic Reviews’, p.1] using selection criteria, at times requiring further information from the nominator, and prioritised according to appropriateness, importance, duplication, feasibility and potential for impact/change 8: ‘Identifying, Selecting and Refining Topics for Comparative Effectiveness Systematic Reviews’, Table 4 p.14]. Topics can be taken forward as systematic reviews or technical briefs; the EHC posts nominations for all topics on its website [2].

AHRQ does not appear to have any entry criteria for tests to be eligible for assessment (such as US FDA approval for example).

### Process for scoping HTA diagnostic topics

The diagnostic topic scope is developed by the EPC report writing team, who the AHRQ advises to solicit input from stakeholders [7, p. 2-2 and 3-5]. Further information relevant to tests is provided in an updated chapter of the General Methods Guide: technical expert groups address various issues to develop all PICOTS parameters of the topic (Population, Intervention, Comparator, Outcome, Timing, Setting), including ‘*What is the potential impact of intervention on patients?*’, ‘*Which outcomes (intended and unintended effects) are relevant, including timing?*’, as well as questions to identify variation in clinical practice. “*Understanding the clinical logic underlying claims about comparative effectiveness is an important goal of topic development*.” [8: ‘Identifying, Selecting and Refining Topics for Comparative Effectiveness Systematic Reviews’, p.15].

Detailed test–specific guidance, alongside examples, is provided in chapters 2 and 3 of the Test Methods Guide [7]. Their central theme is understanding and clarifying the claim of a test [7, p.2-1 to 2-12), and then systematically identifying all important outcomes [7, 3-1 to 3-11].

This process is organised around a set of principles to follow, which starts by using the PICOTS to identify key question components (Population; Intervention (Index test); Comparator; Outcome; Timing; Setting (and study design)) to achieve a conceptual understanding of the details of the question. It is also used to refine the question using stakeholder engagement to arrive at a shared understanding, as well as for operational decisions on arriving at a set of questions to be investigated in the review. *“(1) Use the PICOTS typology to clarify the context relevant to the review, and (2) Use an organizing framework to classify the types of medical test evaluation studies and their relationship to potential key questions*” [7, p.1-8).

Subsequent steps (principles) emphasise the need to understand the “*convoluted linkages between intermediate and health outcomes*” [7, p.2-3], which forms a key philosophy behind AHRQ’s approach to test evaluation. This should be undertaken by constructing question–specific analytic frameworks (also called causal pathways), simple decision trees and using other organizing frameworks. “*Two key components of the analytic framework are: (1) a typology for describing the context in which the test is to be used, and (2) some form of visual representation of the relationship between the application of the test or treatment and the outcomes of importance for decision-making*.” [7, p.1-5].

In certain circumstances, decision trees are recommended as a useful adjunct to the analytical framework, allowing more complex interactions between tests and outcomes to be delineated. They are also suggested as tools to decide whether evidence of test accuracy is sufficient to demonstrate clinical effectiveness, and this should be carefully justified by considering a set of questions listed in the guide [7, p.2-5].

Organising frameworks are the same as conceptual or evaluative frameworks such as the six general aspects of tests which might be identified: technical efficacy, diagnostic accuracy, diagnostic thinking efficacy, therapeutic efficacy, patient outcome, and societal aspects.

“*We suggest that rather than being a hierarchy of evidence, organizational frameworks should categorize key questions and suggest which types of studies would be most useful for the review. They may guide the clustering of studies, which may improve the readability of a review document*.” No specific framework is recommended [7, p.2-6].

Chapter 3 offers principles for addressing the challenges of incorporating all decision-relevant outcomes. Five types of outcomes are identified: clinical management, direct health effects, emotional/social/cognitive/behavioural responses, legal and ethical, and costs. Outcomes should be prioritised, taking into account the needs of stakeholders (given resource constraints) “*who should be assisted in prioritizing the outcomes for inclusion*” [7, p.3-11].

An updated chapter of the General Methods Guide provides further recommendations for considering which harms to evaluate for tests, citing in particular the need to consider overdiagnosis and overtreatment, as well as the immediate and downstream consequences of incorrect diagnosis [8: ‘**Prioritization and Selection of Harms for Inclusion in Systematic Reviews (2018 update)’,** p.11].

2. Review and post–review processes: we looked for information on:

- *What approach is used to develop evidence into guidance? e.g. by use of a committee to consider evidence reviews alongside any consultation processes*
- *How guidance is disseminated*
- *How patients or public, test developers, and/or experts are involved in the review process.*

### Process for involving stakeholders

AHRQ places consultation with stakeholders at the centre of its processes, and it forms part of the AHRQs philosophy and guiding principles:

"*The EHC Program reflects in many ways the decentralized nature of the U.S. health care system. The audience includes not only policymakers in government and private health plans but also clinicians, patients, and members of industry, all of whom play a major role in health care decision–making. All of these stakeholders provide input and guidance to the program, all may contribute suggestions of new topics for assessment, and all have provided comments on drafts of the guidance given in this series. The EHC Program is meant to provide understandable and actionable information for patients, clinicians, and policymakers.*" [8: ‘Comparing Medical Interventions: AHRQ and the Effective Health Care Program (2008)’, p.2)].

As described above, test developers (‘industry’) are involved, although the test methods guide does not clarify to which parts of the process they contribute. The public are able to comment on the topic’s Key Questions and draft report (both its findings and way in which those findings are communicated), by viewing these on the EHC website [4, p.1].

During the editorial review process, the AHRQ seeks comment and review from the public as well as clinical and methodology experts [5, p1].

The AHRQ’s EPC programme uses ‘high stakeholder impact’ to prioritise reviews for update:

“*The stakeholder impact is determined by the utility and uptake of the report as measured by the frequency with which the report or its related products are downloaded, interest from stakeholder partners, and citation of the report in other scientific literature, including clinical practice guidelines.*” [6].

### Processes for developing evidence into guidance and disseminating guidance

The output is the “technology report”, and no separate guidance document is created. These are made freely available, in full, on the organisation’s website.

3. Recommended methods for performing a clinical effectiveness review of a diagnostic test: we looked for guidance on:

- *whether the HTA involves collection of evidence for review of clinical effectiveness;*
- *who collects the information and how this is funded;*
- *which evidence should be sought for tests, and using which search methods;*
- *methods to appraise the quality of evidence, and how to incorporate these into conclusions;*
- *summarising and synthesizing evidence*
- *methods to judge the relative strength and importance of the evidence? (e.g. GRADE)*

### Overview of guidance on methods for Health Technology Assessments of tests

The AHRQ have produced a methods guide specifically tailored for evaluating medical tests [7]. This guide has not been updated since its publication (2012), though more recently updated chapters of the General Methods Guide (for all interventions) [8] contain some methods relevant to test evaluation.

At AHRQ, HTA of tests involves systematic review of any clinical evidence required to inform the assessment of outcomes prioritised during scoping (topic development).

### Methods for reviewing clinical evidence of diagnostic tests

Methods follow the commonly accepted methods for conducting rigorous systematic reviews, with detailed specific tailoring to the evaluation of medical tests.

Detailed guidance for systematically and comprehensively identifying clinical evidence is provided in Chapter 4. As well as following general guidance for any systematic review, the following areas of emphasis are identified: caution not to rely on search filters alone [7, p.4-2]; caution not to rely on controlled vocabulary (subject headings) alone [7, p.4-3]; advice to search in multiple locations with tailored strategies [7, p.4-3]; advice to include all possible variant names of target diagnostic tests, or if not possible to include generic terms for diagnosis [7, p.4-6].

### How should quality be assessed?

Detailed considerations for assessing the quality of diagnostic test accuracy studies are provided in Chapter 5, noting specifically that reviewers should use validated criteria to address relevant sources of bias: “*We recommend that reviewers use criteria for assessing the risk of systematic error that have been validated to some degree from an instrument like QUADAS-2*” [7, p.5-5]. Further, criteria should be applied in a standardised manner, including arriving at decisions on when inadequate reporting constitutes a fatal flaw.

Appraising the quality of ‘end–to–end’ studies should follow methods used for all interventions: “*For trials of tests with clinical outcomes, criteria should not differ greatly from those used for rating the quality of intervention studies*” [7, p.5-4].

Quality assessment of other study designs (for example before–after designs) is not mentioned.

At AHRQ, decisions on which evidence to include should not be solely influenced by the evidence hierarchy (risk of bias), but by the availability of evidence that is applicable to the review topic, and that can address the outcomes that have been prioritised: "*In the Effective Health Care Program, the conceptual model for considering different types of evidence still emphasizes minimizing the risk of bias, but it places high-quality, highly applicable evidence about effectiveness at the top of the hierarchy. The model also emphasizes that simply distinguishing RCTs from observational studies is insufficient because different types of RCTs vary in their usefulness in comparative effectiveness reviews."* [8: ‘Principles in developing and applying guidance’ (2009), p.5]. Detailed test–specific considerations for selecting studies according to the applicability of evidence are provided in Chapter 6 [7], and include guidance to use the PICOTS as framework for identifying important contextual factors.

AHRQ emphasise that a key additional requirement of reviews of tests is that applicability is a direct consequence of the causal chain by which a test impacts downstream outcomes:

“*Decisions should be based on whether there is evidence that a particular contextual factor is expected to influence the performance characteristics of the test or its effectiveness as a component of care.*” [7, p.6-2].

Guidance includes prompts, illustrated with specific examples, to identify elements of this causal chain: “*In an ideal review, all possible factors related to the impact of a test use [sic] on health outcomes should be considered... Consider factors that could affect the causal chain of direct relevance to the key question: for instance, in assessing the accuracy of cardiac MRI for detecting atherosclerosis, slice thickness is a relevant factor in assessing applicability. It is also important to consider applicability factors that could affect a later link in the causal chain (e.g., for lesions identified by cardiac MRI vs. angiogram, what factors may impact the effectiveness of treatment?).*” [7, p.6-6].

Particular focus is given to summarising challenges that are commonly encountered in the applicability of evidence to diagnostic topics: spectrum effects, importance of the role of new tests within existing care, rapidly evolving technology (versions of tests), shifting routine care (comparators) and treatment options (for all diagnostic strategies). “*The root cause of these challenges is that test accuracy, as well as more distal effects of test use, is often highly sensitive to context.*” [7, p.6-3].

### How should evidence be synthesised?

Methods for summarising evidence focus on synthesising test accuracy metrics, with the rationale that this is the most common focus of medical test evaluation, both from a practical and research perspective [7, p.8-2]. Detailed guidance on meta-analytic methods in the presence [7, chapter 8] and absence [7, chapter 9] of a reference standard are provided, alongside numerous references to key methodology texts. We did not find reference to methods for synthesising or summarising evidence from other study designs.

AHRQ has a clear philosophy that reviewing a test’s accuracy is necessary but insufficient to determine the effectiveness of a new test [7, p.8-2], a perspective that is repeated throughout the *Medical Test Methods Guide* by situating ‘accuracy’ as an intermediate outcome which is only indirectly linked to a test’s impact on clinical outcomes: “*Although there is a temptation to use diagnostic accuracy as an intermediate outcome for the effect of a diagnostic test on clinical outcomes, there is often no direct linkage between the diagnostic accuracy outcome and a clinical outcome.*” [7, p.7-3].

Their recommendations do encourage the use of modelling [7, Chapter 10], which some researchers and organisations (not the AHRQ) have referred to as ‘linked evidence’ modelling, to model health outcomes using test accuracy data when there is no evidence of the effect of tests on health outcomes:

“*Modeling (in the form of decision or economic analysis) is a natural framework for linking test performance data to clinical outcomes. We propose that (some) modeling should be considered to facilitate the interpretation of summary test performance measures by connecting testing and patient outcomes.*” [7, p.10-1].

The focus of this chapter is on using modelling to enhance the interpretation of systematic reviews of diagnostic test accuracy:

“*Here we are primarily concerned with a narrower use of modeling, namely to facilitate the interpretation of summary test performance measures by connecting the link between testing and patient outcomes.*” [7, p.10-2]. For example, AHRQ encourage this method to explore possible trade–offs between tests, where one has superior accuracy but the other has a different benefit: “*In case of tradeoffs, i.e., one test has better specificity but another one is safer (with all other attributes being equal), one would have to explore these tradeoffs using modeling.*” [7, p.10-4]. A 5–step process is outlined, although the guidance provides limited detail on how to perform the methods themselves.

### How should the strength of the body of evidence be determined?

Grading the body of evidence on tests is considered in detail in Chapter 7, providing methods for the diagnostic test accuracy evidence base. At AHRQ ‘strength of evidence’ refers to “*the assessment of the strength of the body of evidence supporting a given statement or conclusion, rather than to the quality of an individual study*” [7, p.7-1]. Methods are broadly aligned with the GRADE (Grading of Recommendations, Assessment, Development and Evaluations) approach.

When the body of evidence directly assesses the impact of tests on clinical outcomes, AHRQ recommends that the GRADE principles for interventions can be used or adapted [7, p.7-4].

Grading the strength of an *indirect* body of evidence is identified as a key methodological challenge, for which “*there is no consensus for one particular approach*” [7, p.7-5]. While no method is defined for grading indirect bodies of evidence, AHRQ does identify that a key part of this process should include grading the *links* between included evidence for intermediate outcomes, using the analytic framework as a tool to identify these links:

“*Given that most evidence regarding the clinical value of diagnostic tests is indirect, an analytic framework must be developed to clarify the key questions, and strength of evidence for each link in that framework should be graded separately*.” [7, p.7-1].

“*The strength of the body of evidence regarding the overarching question of whether a test will improve clinical outcomes depends both on the total body of evidence, as well as the body of evidence for the weakest link in this chain.*” [7, p.7-3].

4. Recommended methods for evaluating the cost–effectiveness of diagnostic interventions: we looked for guidance on:

- *whether the HTA should involve any assessment of cost–effectiveness, and if so which methods are recommended (e.g. cost–utility, ‘cost–effectiveness’, cost–minimisation, budget impact analysis);*
- *how evidence should be incorporated into health economic models;*
- *how the clinical effectiveness review and cost–effectiveness study should link together (e.g. the extent to which clinical effectiveness review results are used to inform economic models);*
- *methods for carrying out linked evidence modelling, where this is different from health economic modelling*

### Methods for conducting health economics assessments

Cost–effectiveness is not assessed.

### Special features noted

- Use of the AHRQ ‘analytic framework’ as a lynch pin of reviews. The framework is first developed during topic development (scoping), and is used to provide clarity, consistency and depth to the whole systematic review process. The framework is used to set out the ‘chain’ of outcomes that lead from the test itself to downstream health outcomes.
- “HTA” by Evidence Practice Centers are conceived as systematic reviews of the evidence on tests possibly augmented by modelling. They are not located in a system for making decisions.
- Special attention given to the particular needs of genetic tests [7, Chapter 11] and prognostic tests [7, Chapter 12].

### Questions to AHRQ

1. **Is the content of our summary above a fair and accurate reflection of your organisation’s processes and methods for undertaking (Clinical Effectiveness Reviews of diagnostic tests)?**
2. **Are there any publicly available documents we have missed that outline your processes for undertaking HTAs of tests?** We would like to provide a summary of organisations’ procedures for: topic referrals, pre–HTA scoping/topic development phase, involvement of stakeholders, post–review development of HTA into guidance**.**
3. **How are test developers involved in the CER process?** We have noted that test developers are involved, could you please clarify at which points in the process they are invited to contribute? For example during the topic scoping/question development phase, collection of evidence, or interpretation of evidence.
4. **Are there any publicly available documents we have missed that outline the methods that should be used in an HTA of diagnostic tests?**
5. We are particularly interested in how test accuracy information is translated into conclusions about impact on patients’ outcomes. We note that some people refer to this as evidence linkage or ‘linked–evidence’ modelling. **Your methods imply to us that your use of this modelling is focussed on interpreting diagnostic test accuracy evidence, and that you do not perform modelling to estimate the health impacts of diagnostic tests. Is this correct?**
6. We note that CERs should not involve any estimation of cost–effectiveness. **Is this correct?**

## Canadian Agency for Drugs and Technologies in Health (CADTH)

**Responses from Joanne Kim and Cody Black, on behalf of CADTH.**

### Sources used for information

1. ***About the Health Technology Assessment Service***, CADTH website: <https://www.cadth.ca/about-cadth/what-we-do/products-services/hta> (Last Updated 15th June 2021) [last accessed 16th June 2021].
2. ***Overview of HTA and OU Medical Devices and Clinical Interventions***. [downloaded 24^th^ July 2020]. Ottawa: CADTH.
3. ***Health Technology Assessment and Optimal Use: Medical Devices; Diagnostic Tests; Medical, Surgical, and Dental Procedures.*** CADTH Topic Identification and Prioritization Process. November 2015 (Version 1.0). Ottawa: CADTH. [downloaded 24^th^ July 2020].
4. ***Guidelines for the Economic Evaluation of Health Technologies: Canada.*** ***4th ed.*** Ottawa: CADTH; 2017 Mar. [downloaded 24^th^ July 2020].
5. ***Guidelines for the Economic Evaluation of Health Technologies: Canada. 4th ed. Appendix — Specific guidance for treatments with companion diagnostics.*** Ottawa: CADTH; 2019 Sep. [downloaded 24^th^ July 2020].
6. ***Guidelines for Authors of CADTH Health Technology Assessment Reports.*** Revised May 2003. Ottawa: CADTH. [downloaded 23^rd^ September 2020].
7. ***Health Technology Expert Review Panel***, CADTH website: <https://www.cadth.ca/collaboration-and-outreach/advisory-bodies/health-technology-expert-review-panel> (Last Updated 16th June 2021) [last accessed 16^th^ June 2021].
8. ***Device Advisory Committee***, CADTH website: <https://www.cadth.ca/device-advisory-committee-dac> (Last Updated 16th June 2021) [last accessed 16^th^ June 2021].
9. ***CADTH Health Technology Expert Review Panel Terms of Reference.*** March 2020. Ottawa: CADTH. [downloaded 23^rd^ September 2020].
10. ***CADTH Health Technology Expert Review Panel Deliberative Framework***. May 2015. Ottawa: CADTH.

### HTA setting

Created in 1989 by Canada’s federal, provincial, and territorial governments, the Canadian Agency for Drugs and Technologies in Health (CADTH) provides a coordinated approach to assessing health technologies. It is an organization that harnesses Canadian expertise from every region and produces evidence-informed solutions that benefit patients in jurisdictions across the country.

CADTH is an independent, not-for-profit organization responsible for providing health care decision-makers with objective evidence to help make informed decisions about the optimal use of health technologies, including:

- drugs
- diagnostic tests
- medical, dental, and surgical devices and procedures

In addition to evidence, it also provides advice, recommendations, and tools.

HTA products include:

- *“CADTH Health Technology Assessment — a comprehensive evaluation of the clinical effectiveness, cost-effectiveness, and the ethical, legal, and social implications of health technologies on patient health and the health care system.*
- *Technology Review — the product line used for custom health technology reviews that contain some but not all of the elements of a traditional Health Technology Assessment (for example, an economic review without a clinical component).”* [1]

1. Pre–evidence review processes for HTAs of tests: we looked for information on:

- *Where suggestions for which tests to review come from (referral source)*
- *Whether there are any entry criteria (e.g. regulatory conformity such as CE mark)*
- *Whether a formal scoping phase is recommended or mandated, and if so how the scope should be developed from the referred topic, and what the scope’s focus should be.*

**from the idea that Canada needs a coordinated approach to assessing health technologies. The result was an organization that harnesses Canadian expertise from every region and produces evidence-informed solutions that benefit patients in jurisdictions across the country.**

### Processes for identifying diagnostic topics for HTA reports

There is a robust process for identifying topics and prioritising them, involving 3 stages of assessment and prioritisation. These include diagnostics, and while there is no special process for them some test–specific criteria are provided, including:

- Ensuring the test is approved for use in Canada (Appropriateness criterion, stage 2)
- Prioritising tests that increase equity of healthcare, such as point of care technologies that could be used in remote settings (Prioritization, stage 3) [3].

### Process for scoping HTA diagnostic topics

Again there is a robust general process for scoping all topics, including diagnostics. Following topic identification and prioritisation, detailed scoping is conducted to refine and finalize the decision problem. The decision problem informs which CADTH HTA product will be needed (i.e., a full Health Technology Assessment or a tailored Technology Review) and what research questions should be addressed by CADTH review.

For the health economic model, the following statement describes the considerations for specifying the decision problem and the research questions: “*Specifying a decision problem entails identifying the perspective from which the problem is to be addressed, and specifying the interventions (such as drug treatments, surgical procedures, diagnostic tests) to be compared, as well as the measures (e.g., costs, outcomes) that will be used to compare them.*” [4, p.24].

There is no specific detailed guidance for economic evaluations of diagnostic tests, but occasional test specific issues are mentioned, for example “*When dealing with management strategies, researchers should ensure that uncertainty in the data informing all parts of the strategy is appropriately characterized. For example, if the management strategy consists of both a test and a treatment component, researchers should account for the costs and effects of both false-positive and false-negative test results.*” [4, p.28]

There is some further test relevant advice on defining the decision problem for economic evaluations in the Appendix on Companion Diagnostics [5], particularly on the need to consider ‘diagnostic-therapeutic dependency’, interpretation of test results and changes to clinician behavioural response to results. The importance of the diagnostic setting and place of the test in the management pathway are emphasised: “*There may be several possible ways in which the companion diagnostic can be used within a clinical pathway and/or the results can be interpreted. The decision problem should clearly specify where and when the companion diagnostic will be used in the clinical pathway and how its result will inform the subsequent treatment decision. If there are several possible roles for the companion diagnostic under evaluation, there should be a separate decision problem for each purpose*”. [5, p.7]

2. Review and post–review processes: we looked for information on:

- *What approach is used to develop evidence into guidance? e.g. by use of a committee to consider evidence reviews alongside any consultation processes*
- *How guidance is disseminated*
- *How patients or public, test developers, and/or experts are involved in the review process.*

### Process for involving stakeholders

CADTH outline a general system for involvement of stakeholders throughout the HTA process, which also applies to tests. This is illustrated by statements such as:

For the Device Advisory Committee, roles and responsibilities include: “*To participate in the CADTH topic identification and prioritization process*” [8, p.1]

“*It is good practice to specify the decision problem in consultation with clinicians, members of the target population, and the decision-maker(s) to ensure that all relevant comparators are included; the most relevant outcomes for each stakeholder are taken into account; and the assessment is founded on a thorough understanding of all available evidence*” [4, p.25], and

“*Before being released, HTA products are peer-reviewed by external clinical, economic, and methodological experts as appropriate and internal CADTH staff.*” [1].

Clinical experts are key members of the review process, engaged as either a co-author or a peer-reviewer, to assist in the development of the topic, research questions, and protocol and to review the draft report. Clinical experts are also key members of the recommendation process, serving on HTERP to produce final guidance [7].

Similarly, CADTH involves patients, patient families, and patient groups in reviews, and a public member and a patient member serve on HTERP [8].

### Processes for developing evidence into guidance and disseminating guidance

Not all HTA projects receive recommendations from HTERP.[2] For HTA projects that require recommendations, known as ‘Optimal Use’ HTA products, the Health Technology Expert Review Panel [9] considers the evidence review report developed by CADTH and makes recommendations using a multicriteria ‘deliberative framework’ [10]. This has no test specific components. Recommendations are by consensus or majority vote [9, p.3].

3. Recommended methods for performing a clinical effectiveness review of a diagnostic test: we looked for guidance on:

- *whether the HTA involves collection of evidence for review of clinical effectiveness;*
- *who collects the information and how this is funded;*
- *which evidence should be sought for tests, and using which search methods;*
- *methods to appraise the quality of evidence, and how to incorporate these into conclusions;*
- *summarising and synthesizing evidence*
- *methods to judge the relative strength and importance of the evidence? (e.g. GRADE)*

### Overview of guidance on methods for Health Technology Assessments of tests

Clinical effectiveness reviews are discussed as part of informing parameters in the health economic model.

### Methods for reviewing clinical evidence of diagnostic tests

There are no publicly available methods guidelines for performing a clinical effectiveness review for CADTH HTA products. However, “*The work is carried out by a multidisciplinary team of researchers whose composition is appropriate to the nature of the project. Before being released, HTA products are peer-reviewed by external clinical, economic, and methodological experts as appropriate and internal CADTH staff. The findings are summarized in reports that translate the scientific and economic data into information relevant to decision-making.*” [1].

There is no reference to specific methods for clinical effectiveness reviews of tests. However, a past HTA project states using DTA-relevant resources such as the Cochrane Handbook for Systematic Reviews of Diagnostic Test Accuracy and the QUADAS-2 instrument.

The appendix on companion diagnostics briefly mentions:

“*Evidence of companion diagnostic and treatment performance should be selected in a comprehensive and unbiased manner, using appropriate methods of systematic literature review*.” [5, p.10], and:

“*Where a meta-analysis of accuracy data is undertaken to produce evidence to parameterize the model, adherence with best practice in the statistical methods used (as outlined in the Cochrane Handbook for Diagnostic Test Accuracy Reviews) is required*.” [5, p.11]

The importance of ensuring that populations in the research are similar to those where the companion diagnostic will be used is also emphasised: “*Linking evidence is meaningful when the evidence for the proposed companion diagnostic(s) and for the proposed treatment(s) has been generated in similar patient populations that reflect the target population.*” [5, p.10].

4. Recommended methods for evaluating the cost–effectiveness of diagnostic interventions: we looked for guidance on:

- *whether the HTA should involve any assessment of cost–effectiveness, and if so which methods are recommended (e.g. cost–utility, ‘cost–effectiveness’, cost–minimisation, budget impact analysis);*
- *how evidence should be incorporated into health economic models;*
- *how the clinical effectiveness review and cost–effectiveness study should link together (e.g. the extent to which clinical effectiveness review results are used to inform economic models);*
- *methods for carrying out linked evidence modelling, where this is different to health economic modelling*

### General approach

There is detailed guidance on the general approach to economic evaluation, but no specific recommendations for economic evaluation where the technology is a test.

There is some specific guidance for economic evaluations on approach where tests are used as “companion diagnostics”. Where end-to-end evidence is not available, a “linked evidence approach” can be used: “*When there are no clinical studies that follow patients from diagnosis to downstream health outcomes (i.e., clinical utility studies of the companion diagnostic), different types of evidence can be linked together via decision-analytic modelling.*” [5, p.10].

No detail on how to perform linked evidence analysis is provided, however CADTH note that: “*Linking evidence is meaningful when the evidence for the proposed companion diagnostic(s) and for the proposed treatment(s) has been generated in similar patient populations that reflect the target population.*” [5, p.10].

There is a specific acknowledgement that multiple factors influence a diagnostic and treatment decision (aside from a test’s result), with a requirement for authors to justify any assumption that diagnostic results are the single driving factor in clinical decision–making [5, p.10].

### Detailed methods for evaluating the cost-effectiveness of diagnostic tests

A cost-utility analysis (CUA) is the recommended type of economic evaluation and should be used in the reference case analysis, with outcomes expressed as QALYs. [5, p.12]

The guidelines for economic evaluations are generic, with little specific to any intervention. Occasional examples are offered in parentheses, for example ‘Selecting Comparators’: “*In some cases, comparators may be management strategies…When dealing with management strategies, researchers should ensure that uncertainty in the data informing all parts of the strategy is appropriately characterized. For example, if the management strategy consists of both a test and a treatment component, researchers should account for the costs and effects of both false-positive and false-negative test results.*” [4, p.28].

Areas requiring additional emphasis and attention are identified in the appendix on companion diagnostics, for instance:

- All key clinical outcomes and costs should be accounted for in the reference case, including for example clinician or patient adherence, key clinical outcomes from treatment and diagnosis, and differences in complications and complication rates [5, p.7–8].
- The need to consider all possible comparators, including ‘no test/no treatment’ and ‘no test/treat everyone’, as well as alternative diagnostics. [5, p.8]
- Advice to collect evidence on the effectiveness for all interventions within the companion–diagnostic: “*All evidence for each identified relevant clinical pathway to be compared should be identified.*” [5, p.9]
- Specific considerations for incorporating evidence of test accuracy addressing: selecting the right population [5, p.9]; selecting appropriate reference standard(s) [5, p.10]; performance data at different accuracy thresholds [5, p.10–11]; modelling consequences to false positive and false negative patients [5, p.11]; incorporating incidental findings and changes in case–mix when modelling multiple test scenarios [5, p.11].

Concerning considerations for uncertainty, the appendix on companion diagnostics highlights that models should incorporate a list of ‘scenario analyses’ as standard, which should include the prevalence of individuals that should receive treatment, proportion of patients eligible for testing, uptake rate of the test, diagnostic accuracy, treatment effectiveness, diagnostic impact (“*heterogeneity in clinical and/or patient behavioural response to companion diagnostic results*”) and the impact of clinician and patient preferences [5, p.13]. Recognising that the evidence–base for companion diagnostics is likely to be less well developed than for pharmaceutical interventions, the appendix on companion diagnostics states: “*As a result, decision-makers may be particularly interested in the value of gathering additional evidence on different components of the decision problem as well as the expected cost-effectiveness of the technology given the current evidence base. Therefore, analysts should consider the provision of expected value of partial information (EVPI) analyses….*” [5, p.13].

### Special features noted

None

### Questions to CADTH

**We would like to ask you 7 questions regarding the CADTH approach for conducting HTAs of tests, and would be happy to discuss by telephone/zoom, or via email:**

1. **Is the content of our summary above a fair and accurate reflection of your organisation’s processes and methods for undertaking HTAs of diagnostic tests?**

Thank you for the opportunity to review this summary. We have added and tracked changes and comments throughout the summary above for an accurate reflection.

1. **Are there any publicly available documents we have missed that outline your processes for undertaking HTAs of tests?** We would like to provide a summary of organisations’ procedures for: topic referrals, pre–HTA scoping/topic development phase, involvement of stakeholders, post–review development of HTA into guidance**.**

We have provided relevant references throughout the summary above.

1. **Are test developers (‘industry’), patients and experts involved in any other part of the HTA process?** We have noted that these stakeholders are involved in various stages of producing CADTH reviews and guidance. Have we missed any other stages that they are involved in?

We have made some clarifications and additions on this above.

1. We could not find any guidance for conducting clinical effectiveness reviews. **Are there any publicly available documents we have missed that outline the methods that should be used in an HTA of diagnostic tests?** Also, methods for economic evaluations are a prominent feature of your guidance, **have we missed anything on how to adapt general economic evaluative approaches to tests?**

No, we do not have any publicly available guidance for conducting clinical effectiveness reviews of diagnostic tests. No, we do not believe anything relevant to economic evaluations was missed.

1. We note that you provide specific guidance on the economic evaluation of tests in the Appendix on “Companion Diagnostics”. **Is it intended that this guidance is only to be used in Companion Diagnostics, or could it be used for other types of diagnostics?**

It is intended to be used only in companion diagnostics as defined on Page 5 of the document. Further, as stated on Page 6 of the document: *“For the purpose of this guidance, the definition of a companion diagnostic is limited to a test that is used to identify individuals who are most likely to benefit or experience harms from a defined therapeutic intervention. Other scenarios, such as the use of a companion diagnostic to monitor therapeutic response or a comprehensive assessment of the companion diagnostic (when there are multiple potential uses of the companion diagnostic), are beyond the scope of this guidance.”* While the guidance may also be applicable to other types of diagnostics, that would depend on each diagnostic or each type of diagnostics.

1. We are particularly interested in how test accuracy information is translated into conclusions about impact on patients’ outcomes. **We note that CADTH recommends linked–evidence modelling for the purposes of cost–effectiveness evaluation, and do not perform modelling to estimate the effectiveness of diagnostic tests. Is this correct?**

It depends on what clinical evidence is available. We believe the following text from Page 9 of Ref [5] clarifies this point: *“Clinical effects should be obtained from the specific population being considered (e.g., effectiveness of treatment for true-positive patients should be gleaned from populations who test positive and receive treatment, and not just studies considering treatment alone without testing). Similarly, the effects for false positive patients should be reflective of the specific population. In the absence of linked companion diagnostic-treatment effectiveness data, explicit reporting of the patient population from which included effectiveness data has been obtained, and how it relates to the characteristics of the population affected by the decision, is required. The implications of the uncertainty attributable to using treatment only effectiveness estimates must be described.”* In other words, the order of preferred evidence is: 1) direct evidence from clinical studies that followed patients from diagnosis to downstream health outcomes; 2) linkage of evidence for the proposed companion diagnostic(s) with evidence for the proposed treatment(s) that has been generated in similar patient populations; or 3) in the absence of the previous two, explicit reporting of the patient population from which included effectiveness data has been obtained and how it relates to the characteristics of the population affected by the decision.

The section titled “Linking Evidence via Decision-Analytic Modelling” on Page 10 of Ref [5] provides further relevant details.

1. We are also particularly interested in how HTA organisations identify the claims of diagnostic tests, which we note are sometimes referred to as ‘value propositions’ or ‘proposed benefits and harms’. **Do you have a particular process or approach for determining the claims of a diagnostic test selected for HTA?**

At CADTH, we consider all components of an HTA project in determining the benefits and harms of a health technology. This is reflected in our review process (see Ref [2] for the various components of a full HTA) as well as the recommendation process (see Ref [10] for the various domains of the HTERP deliberative framework). As such, we suggested deleting text above that described economic evaluations as the main focus of our HTA projects and as the main means of deciding the value of a new technology.

## Institute for Quality and Efficiency in Health Care (Institut für Qualität und Wirtschaftlichkeit im Gesundheitswesen (IQWiG, Germany)

### Sources used for information

1. **General methods** **(Version 5.0, 10^th^ July 2017).** English translation of *Allgemeine Methoden*. (Available from: <https://www.iqwig.de/en/about-us/methods/methods-paper/>). [pdf downloaded 24^th^ July 2020].
2. **General Methods (Version 6.0, 5th Nov 2020).** Informal translation of Final Draft of *Allgemeine Methoden*. (Available from: <https://www.iqwig.de/en/about-us/methods/methods-paper/>). [pdf downloaded 12^th^ December 2020].
3. **Basic principles.** (Available from: <https://www.iqwig.de/en/methods/basic-principles.3314.html>]. (Last accessed 24^th^ July 2020).
4. **Contracting agencies and funding of IQWiG.** [available from: <https://www.iqwig.de/en/about-us/responsibilities-and-objectives-of-iqwig/contracting-agencies-and-funding.2951.html>] (Last accessed 24^th^ July 2020).
5. **Institute structure.** (Available from: <https://www.iqwig.de/en/about-us/institute-structure.2953.html>). (Last accessed 24^th^ July 2020).
6. **About us – An introduction to IQWiG.** (Available from [www.IQWiG.de](http://www.IQWiG.de)). [pdf downloaded 24^th^ July 2020].
7. **Non–drug interventions: responsibilities of the department.** (Available from: <https://www.iqwig.de/en/about-us/institute-structure/departments/non-drug-interventions.2976.html>). (Last accessed 24^th^ July 2020).

### HTA setting

Health technology assessment (HTA) in Germany is led by its national body, the Institute for Quality and Efficiency in Health Care (IQWiG), which was founded in the course of the health reform in 2004 as an institution of the Foundation for Quality and Efficiency in Health Care. Its tasks are [1]:

- Research, presentation and evaluation of the current state of medical knowledge on diagnostic and therapeutic procedures for selected diseases.
- Creation of scientific reports, expert reports and opinions on questions of under the statutory health insurance the quality and efficiency provided services under consideration age, gender and life-layer-specific features.
- Assessment of evidence-based guidelines for the epidemiologically most important diseases.
- Making recommendations on disease management programs.
- Assessment of the benefits and costs of drugs.
- Provision of general information that is understandable for all citizens on the quality and efficiency of health care as well as on the diagnosis and treatment of diseases with significant epidemiological importance.
- Participation in international projects for cooperation and development in the field of evidence-based medicine.

This encompasses Health Technology Assessment (HTA) as we have defined it.

1. Pre–evidence review processes for HTAs of tests: we looked for information on:

- *Where suggestions for which tests to review come from (referral source)*
- *Whether there are any entry criteria (e.g. regulatory conformity such as CE mark)*
- *Whether a formal scoping phase is recommended or mandated, and if so how the scope should be developed from the referred topic, and what the scope’s focus should be.*

### Processes for identifying diagnostic topics for HTA reports

Topics are generally referred to IQWiG by the Federal Joint Committee (G-BA) or Federal Ministry of Health, and can be sourced from industry, healthcare providers or the public. External applicants (such as device manufacturers or hospitals) can apply for evaluation by submitting meaningful documents to the G-BA; IQWiG selects from these proposals (1, p.29; 2, p.3).

*“In IQWiG’s ‘Themencheck Medizin’ (Topic check medicine) members of the public can propose medical topics for the assessment of examination and treatment procedures. If a proposal refers to an urgent and still open medical question, a so-called HTA report is produced in collaboration with external researchers.*’ [3]

There is an internal process, including “Dossier assessment”, in G-BA prior to referral. IQWiG assists with the dossier assessment process [1][2].

### Process for scoping HTA diagnostic topics

International standards of Evidence–Based Medicine (EBM) are applied. After commissioning by the G-BA or Federal Ministry of Health, the research question is formulated. This is further developed in the research plan which is consulted on and modified in response to comments received. The importance of current diagnostic practice is noted, with recognition of triage and add-on as specific ways a new test may be incorporated into existing testing pathway. No special considerations for scoping of test topics are mentioned beyond this.

2. Review and post–review processes: we looked for information on:

- *What approach is used to develop evidence into guidance? e.g. by use of a committee to consider evidence reviews alongside any consultation processes*
- *How guidance is disseminated*
- *How patients or public, test developers, and/or experts are involved in the review process.*

### Process for involving stakeholders

Stakeholder involvement is integral to the IQWiG HTA process: “*The law obliges the institute to employ technical experts, manufacturers and the organizations responsible for the protection of the interests of patients and the self-help of chronically ill and disabled people, as well as the representatives, in all important sections of the report to give the federal government the opportunity to comment on the interests of patients. The institute goes beyond this obligation by giving all interested persons and institutions the opportunity to comment on its reports*.” [1, p.4; 2, p.4].

Test developers are not directly involved in the creation of HTAs, but are given the opportunity to comment on the draft report [1, p.40]. Manufacturers can submit applications during the “Assessment of potential” process, and are permitted/requested to submit dossiers for health economics review.

### Processes for developing evidence into guidance and disseminating guidance

The IQWiG report is consulted on and then used by G-BA to make a decision on reimbursement. According to the law, the G-BA must consider these recommendations [in IQWIG report] in its decision-making processes.

There is a clear statement on recommendations in the absence of suitable evidence:

“If it emerges that studies of the required quality and precision are generally lacking, it is the core task of the Institute to describe the circumstances and conclude that on the basis of the “currently best available” evidence, it is not possible to make reliable recommendations” [1, p.8; 1, p.8]. The report and its intermediate stages are made freely available, with final report summaries also produced in English.

3. Recommended methods for performing a clinical effectiveness review of a diagnostic test: we looked for guidance on:

- *whether the HTA involves collection of evidence for review of clinical effectiveness;*
- *who collects the information and how this is funded;*
- *which evidence should be sought for tests, and using which search methods;*
- *methods to appraise the quality of evidence, and how to incorporate these into conclusions;*
- *summarising and synthesizing evidence*
- *methods to judge the relative strength and importance of the evidence? (e.g. GRADE)*

### Overview of guidance on methods for Health Technology Assessments of tests

The general approach is laid out in the General Methods guide, which was recently updated in November 2020 [2]. Both the prior edition [1] and current edition contain special considerations relevant to tests:

**3.5** Diagnostic tests

**3.6** Early diagnosis and screening

At IQWiG, HTA of tests involves systematic review of the clinical evidence, to international standards of EBM. There is an emphasis on restricting studies for inclusion based on quality and study design.

### Methods for reviewing clinical evidence of diagnostic tests

For searching, international standards of EBM are applied as detailed in Chapter 8 [1,2]. No special considerations for tests are mentioned, except noting uncertainty about search filters for accuracy studies.

For study inclusion: “the Institute therefore determines beforehand which study types can be regarded as feasible on the basis of the research question posed, and provide sufficient certainty of results (with high internal validity). Studies not complying with these minimum quality standards (see also Section 9.1.4) are not given primary consideration in the assessment process.” [1, p.60].

IQWiG places strong emphasis on studies capturing patient benefit, and across all intervention types (including tests) the main focus is on RCTs:

“*Prospectively planned controlled diagnostic studies of phase 4 according to Köbberling et al. or studies of level 5 according to Fryback and Thornbury with an (ideally random) allocation of patients to a strategy with or without application of the diagnostic measure to be tested…. are also assigned the highest level of evidence*” [1, p.66; 2, p.70].

“*The Institute follows this logic and primarily conducts benefit assessments of diagnostic tests on the basis of studies designed as described above [RCTs] that investigate patient-relevant endpoints*.” [1, p.66; 2, p.70–71]

Test accuracy evidence on its own is generally not considered:

“*Overall, it is less decisive to what extent diagnostic or prognostic information can determine a current or future state of health*.” [1, p.67; 2, p.71].

“*The mere acquisition of diagnostic information (without medical consequences) generally has no socially relevant benefit*.”……‘ [1, p.66; 2, p.70].

An exception is noted where there is demonstration of equivalent accuracy and where the test has less direct harms than current tests:

“*To demonstrate benefit, in these cases test accuracy studies could be sufficient in which it is shown that the test result of the previous test (= reference standard) and that of the test under investigation (= index test) are identical in a sufficiently high proportion of patients (one-sided question of equivalence)*.” [1, p.68; 2, p.73.]

“*For a comparison of 2 or more diagnostic tests with regard to test quality properties, the highest certainty of results from cohort and cross-sectional studies in which the diagnostic tests are carried out independently of one another on the same patients and are mutually blinded*”. [1, p.68; 2, p.73].

There is also caution on use of Fryback & Thornbury level 3 or 4 evidence on impact on decision making):

“*Information on management changes alone cannot therefore be drawn upon to provide evidence of a benefit, as long as no information on the patient-relevant consequences of such changes is available*.” [1, p.70; 2, p.74].

The importance of comparing tests is highlighted:

“*If a study is to provide informative data on the benefit, diagnostic quality or prognostic value, it is essential that a comparison is made with the previous diagnostic procedure. Only in this way can the added value of the diagnostic or prognostic information be reliably determined*.” [1, p.69; 2, p.73]

Also the role of tests in the clinical pathway:

“*It is also conceivable that a new diagnostic method should be embedded in an already existing diagnostic strategy, for example in such a way that a new test should precede (triage test) or follow it (add-on test), to reduce the use of each other*.” [1, p.70; 2, p.74.]

Concerning quality assessment, international standards of EBM are applied detailed in Chapter 9.1 [1, p.162–168; 2, p162–169]. There is a standard approach to quality assessment of RCTs [1, p165–166; 2, p.166–167]; QUADAS-2 is used for accuracy studies noting that it should be adapted to each specific project [1, p.69; 2, p.74]; the PROBAST instrument is suggested for methodological evaluation of prognostic studies [1, p.69; 2, p.74].

Concerning analysis international standards of EBM are applied detailed in Chapter 9 [1, 2] with separate approaches to the meta-analysis of accuracy data being required [1, p.183-4].

4. Recommended methods for evaluating the cost–effectiveness of diagnostic interventions: we looked for guidance on:

- *whether the HTA should involve any assessment of cost–effectiveness, and if so which methods are recommended (e.g. cost–utility, ‘cost–effectiveness’, cost–minimisation, budget impact analysis);*
- *how evidence should be incorporated into health economic models;*
- *how the clinical effectiveness review and cost–effectiveness study should link together (e.g. the extent to which clinical effectiveness review results are used to inform economic models);*
- *methods for carrying out linked evidence modelling, where this is different to health economic modelling*

International standards of health economics are applied detailed in Chapter 4 [1, 2]. Health economic modelling of cost-utility (cost per QALY) is the main approach. No modification or special consideration for tests mentioned. It is not clear how widely health economics assessment for tests is employed.

### Special features noted

- Strong preference for evidence based on RCTs even for tests, and that an absence of such evidence should result in a recommendation that it is not possible to make reliable recommendations.

### Questions to IQWiG

1. **Is the content of our summary above a fair and accurate reflection of your organisation’s processes and methods for undertaking HTAs of diagnostic tests?**
2. **Are there any publicly available documents we have missed that outline your processes for undertaking HTAs of tests?**
3. **We note that for tests the pre–HTA process involves “Assessments of Potential” (either standard or as high–risk devices) [1, p.26–27]. Is this a preliminary report on the clinical effectiveness of tests?**
4. **We note that test developers are not directly involved in contributing to the HTA, beyond commenting on the draft report. Could you confirm this is the case, or whether they also contribute to the topic scoping/question development phase, collection of evidence, interpretation of evidence, or production of final guidance?**
5. **Are there any publicly available documents we have missed that outline the methods that should be used in an HTA of diagnostic tests?**
6. We are particularly interested in how test accuracy information is translated into conclusions about impact on patients’ outcomes. We note that some people refer to this as evidence linkage or ‘linked–evidence’ modelling. **Your methods imply to us that you accept only direct evidence of clinical effectiveness (measured in a clinical study), and do not perform modelling to estimate the health impacts of diagnostic tests when your evidence–base is restricted to diagnostic reliability studies. Is this correct?**
7. We are also particularly interested in how HTA organisations identify the claims of diagnostic tests, which we note are sometimes referred to as ‘value propositions’ or ‘proposed benefits and harms’. **Do you have a particular process or approach for determining the claims of a diagnostic test selected for HTA?**
8. **Are health economic evaluations also used for tests? If they are, could you confirm that there are no particular modifications or special considerations applied for tests?**

## Medical Services Advisory Committee (MSAC, Australia)

**Sources used for information:**

1. Medical Services Advisory Committee. 2021. *Guidelines for preparing assessments for the Medical Services Advisory Committee*. [Online]. Version 1.0. [Last accessed 16^th^ June 2021]. Available from: <http://www.msac.gov.au/internet/msac/publishing.nsf/Content/Documents-for-Applicants-and-Assessment-Groups>
2. Medical Services Advisory Committee (MSAC) Reform Implementation. 2016. *Process Framework*. [Online]. Version 1.0. [Last accessed 20^th^ July 2021]. Available from: <http://www.msac.gov.au/internet/msac/publishing.nsf/Content/msac-process-framework>
3. Australian Government Department of Health. 2021. *Application Form*. [Online]. Version 2.4 [Last accessed 7^th^ July 2021]. Available from: <http://www.msac.gov.au/internet/msac/publishing.nsf/Content/Documents-for-Applicants-and-Assessment-Groups>
4. Australian Government Department of Health. 2021. *MSAC Application Form Instructions*. [Online]. Version 1 [Last accessed 7^th^ July 2021]. Available from: <http://www.msac.gov.au/internet/msac/publishing.nsf/Content/Documents-for-Applicants-and-Assessment-Groups>
5. Australian Government Department of Health. 2021. *PICO Confirmation (To guide a new application to MSAC)*. [Online]. Version 2.0 [Last accessed 7^th^ July 2021]. Available from: <http://www.msac.gov.au/internet/msac/publishing.nsf/Content/Documents-for-Applicants-and-Assessment-Groups>
6. Medical Services Advisory Committee. 2021. *ADAR Assessment Report Template*. [Online]. 19^th^ May 2021 [Last accessed 7^th^ July 2021]. Available from: <http://www.msac.gov.au/internet/msac/publishing.nsf/Content/Documents-for-Applicants-and-Assessment-Groups>
7. Medical Services Advisory Committee. 2021. *DCAR Assessment Report Template*. [Online]. 19^th^ May 2021 [Last accessed 7^th^ July 2021]. Available from: <http://www.msac.gov.au/internet/msac/publishing.nsf/Content/Documents-for-Applicants-and-Assessment-Groups>
8. Medical Services Advisory Committee. 2021. *ADAR Commentary*. [Online]. 19^th^ May 2021 [Last accessed 7^th^ July 2021]. Available from: <http://www.msac.gov.au/internet/msac/publishing.nsf/Content/Documents-for-Applicants-and-Assessment-Groups>
9. Medical Services Advisory Committee. (Page last updated 21^st^ May 2021). MSAC Membership [Online]. Available from: <http://www.msac.gov.au/internet/msac/publishing.nsf/Content/msac-membership>) [Last accessed 12^th^ July 2021].

**HTA setting:**

Health technology assessment (HTA) in Australia is led by two national, independent bodies who each have a remit for different healthcare technologies. Medicines and vaccines are evaluated by the Pharmaceutical Benefits Advisory Committee (PBAC). The Medical Services Advisory Committee – MSAC provides HTA-based advice on the funding of ‘health technologies other than medicines’ which include investigative, surgical or medical procedures, other programs (blood products or screening programmes), highly specialised therapies delivered as state–based services, and services provided with prostheses [1, p.10]. Investigative services include all types of medical testing, defined as: “*a service that generates clinically-relevant information about the individual to whom the service is rendered*” [4, p.8], and including those used for screening, diagnosis and staging, monitoring, predisposition, prognosis and prediction, as well as imaging and genetic tests [1, p.17].

MSAC is a nonstatutory committee established by the Australian Minister for Health in 1998, whose role it is to provide recommendations to the Australian Health Ministry on the public funding of proposed (new or amended) health services [2, p.9]. At MSAC HTA reports are termed ‘assessment reports’ and can be performed both by either the applicant or an external HTA group contracted on its behalf (Applicant–developed assessment reports, ADARs) [6], or alternatively MSAC can contract an external HTA group directly (Department–contracted assessment reports, DCARs) [7]. The decision on which path to follow is informed by the applicant’s ability to undertake and engage in the HTA process [2, p.42]. External HTA groups also prepare commentaries of ADARs for consideration by MSAC [8].

1. Pre–evidence review processes for HTAs of tests: we looked for information on:

- *Where suggestions for which tests to review come from (referral source)*
- *Whether there are any entry criteria (e.g. regulatory conformity such as CE mark)*
- *Whether a formal scoping phase is recommended or mandated, and if so how the scope should be developed from the referred topic, and what the scope’s focus should be.*

**Processes for identifying diagnostic topics for HTA reports:**

Proposals for MSAC’s consideration can include applications for new and amended requests for public funding of medical services [3,4] and come from various sources, including the medical profession, medical industry, or others seeking Australian Government funding [4, p.4].

MSAC specifies two entry criteria for consideration of medical services applications for funding under Australia’s Medicare arrangements: that they must be a clinically relevant professional service (generally accepted by the medical profession as necessary for the appropriate treatment of patients), and that they match current government policy for funding public health services [4, p.4].

MSAC has developed a detailed *Process Framework* which comprehensively sets out the decision–making processes involved in selecting applications for review, as well as the processes needed to produce the final HTA report [2].

A triaging process takes place before scoping, in which the availability of evidence and the suitability of the application for evaluation through the MSAC framework are assessed [2, p.15]. Criteria for making these decisions are described in detail, to ensure processes are standardised and consistent.

A key component of the application form is setting out the ‘clinical claims’ of the health technology, for which detailed guidance is provided [1, TG1.2, p.21–29]. Claims for any technology must relate to the health of patients, by comparison to existing/alternative practice:

“*Appropriate clinical claims for health outcomes are:*

- *The use of the proposed technology results in superior health outcomes compared to the comparator/standard practice.*
- *The use of the proposed technology results in noninferior health outcomes compared to the comparator/standard practice.*
- *The use of the proposed technology results in inferior health outcomes compared to the comparator/standard practice*.” [1, p.22].

Tests are specifically considered: “*The clinical claim can be informed by understanding the benefits of the test (relative to current care), and how this is likely to affect patient management, and ultimately patients’ health outcomes (see Table 1). All health technologies, including investigative technologies (tests), are required to establish a claim that relates to health outcomes*.” [1, p.23].

Detailed explanation is provided for constructing the clinical claims of tests, with clear guidance that the nature of a test’s claim is derived by comparison with another (generally standard care) test (‘comparative function’), relies on its comparative role (replacement, add–on, triage), and can affect health outcomes through its ability to change a patient’s management. Possible benefits of tests are cross–tabulated according to the types of comparison, effect on patient management and health outcomes, with guidance suggesting the broad category of supporting evidence required [1, Table 1, p.24–5].

Specifically for tests only, MSAC allows additional claims to be proposed. These are conceived as benefits not related to the health outcomes for patients (‘value of knowing’), but which could justify an increase in cost when a clinical claim of noninferiority is proposed, or when the health benefits of a clinical claim of superiority would not otherwise justify the increase in cost [1, p.28–29]:

“*the value of knowing encompasses any consequence for the wellbeing of a patient beyond the changes in the health outcomes attributed to changes in the health care provided. These additional outcomes may or may not be able to be demonstrated with quantitative data. The benefits or harms for people other than the patient receiving the health technology (e.g. their family members or carers) is also considered.”*

*“Value may still be derived from the test results if they result in, for example, avoiding a lengthy diagnostic odyssey, being able to plan for end of life, engagement with others with a similar diagnosis, or financial support being available because the diagnosis allows access to disability schemes.”* [1, p.222].

A notable feature is MSAC’s use of a ‘hierarchy of claims’ to decide on the appropriateness of a claim which places evidence of improvements to health outcomes (‘superiority claim’) at the apex, followed by: evidence of no change in health outcomes (noninferiority), value of knowing claims, tests that do not alter clinical practice, and at the bottom tests whose main claim is the provision of reassurance [1, Table 2, p.26–7]. Tests that detect heritable mutations, and so change family planning options, are also included as a specific claim which MSAC considers more important than ‘value of knowing’ claims.

**Process for scoping HTA diagnostic topics:**

The pre-assessment stage of the MSAC process includes a formal scoping phase, termed ‘PICO Confirmation’, for which a template is provided [5]. The Australian Government’s Department of Health engages an external HTA group to develop the PICO confirmation [2, p.42].

MSAC places a strong emphasis on clearly articulating the PICO and developing related review questions: “*the development of the PICO and assessment questions is a pivotal part of the development of an assessment report.*” [1, p.19].

The resulting document is generally examined by a subcommittee to ensure it is adequate: “*The MSAC application process may involve different pathways. For most applications that progress to an assessment report, the PICO Advisory Sub-Committee (PASC) first considers a PICO confirmation to focus the assessment report and ensure it is clinically relevant*.” [1, p.19].

The focus of the document is threefold:

- to define the proposed PICO (population targeted, intervention test, comparator test(s) and key outcomes),
- to set out the comparative patient management algorithms or flowcharts graphically and by description of the differences between them, and
- for tests, to define a reference standard for the purposes of test accuracy analysis [1, p.30].

Detailed direction is provided to prepare the comparative management flowcharts, with guidance to use literature review, expert panel and expert opinion, as well as on which components of care to include [1, p.41–2]. These flowcharts should be developed from the PICO, “*and should inform the structure of the economic model*” [1, p.41].

MSAC includes numerous test–specific considerations for each element of the PICO. Primarily, MSAC emphasises that each PICO inherently reflects a single intended use of a test, advising that additional PICOs are required if the study test has multiple intended purposes (e.g. diagnosis and monitoring) [1, p.30], multiple intended target populations [1, p.30] or multiple comparators [1, p.37].

Population: guidance directs reviewers to also consider “*populations not receiving the health technology*” when a key claim of the test relates to its impact on broader society (e.g. impact of detecting infectious disease) or a patient’s family members/carers [1, p.33]. Special additional considerations are noted for codependent technologies and genetic tests [1, p.32–33].

Intervention: MSAC provides detailed guidance for describing the diagnostic setting, focussing on how a proposed test would be incorporated into an existing pathway including: the role of the test (add–on, replacement, triage), any other elements of the test strategy, how the test is ordered, how the test is performed, how results are interpreted (including cut–off points), how the result is communicated to the tested population, and how the result is used to inform clinical decisions [1, p.33]. MSAC highlights:

“*As the health benefit of a test is indirect (i.e. it only influences health indirectly through the information provided by the test results), describe the downstream consequences of the proposed test that support the clinical claim (e.g. if a health benefit is claimed, how the benefit is achieved, and what treatments follow positive test results and negative test results*).” [1, p.33].

“*Identify contextual factors that could modify the effectiveness, clinical utility, test accuracy or safety of the health technology (such as the ‘learning curves’ of service providers). Discuss whether there are likely to be any implementation issues (e.g. a change in the specialty that delivers the technology, sample storage requirements, education and training requirements, changes in access to care, communication between and within organisations). Provide details of any quality assurance program or training program already in place or required*.” [1, p.34].

“*If the technology is investigative, discuss whether the biomarker is correlated with factors such as ethnicity and sociodemographic status, and whether there are therefore ethical considerations that must be considered*.” [1, p.34]

Specific requirements are given for tests that are ‘multifactorial algorithms’ [1, p.34–5], e.g.: “*For a fixed algorithm, the dataset that was used for training the algorithm should be clearly described, including whether it was a convenience sample consisting of some positive and negative cases (diagnostic case control), or whether the tested cohort represents the characteristics of the target population in real-life practice*.”. Specific requirements for genetic tests are also provided [1, p.35].

Comparator: Test–specific guidance for selecting the comparator emphasises the need to consider how the new test intends to be added to current patient management (i.e. by replacing or adding to existing testing). Key requirements are that the cost–effectiveness of the comparator should be known or established within the review [1, p.36], and that the comparator: “*should be clearly identifiable in the clinical management algorithm*.” [1, p.37].

The importance of carefully specifying comparator tests is highlighted: “*Avoid comparing the proposed health technology against a ‘basket’ of comparators, where the effect of the proposed technology against individual comparators cannot be derived*.” [1, p.37].

Reference standard: Standard guidance is provided for selecting a reference standard [1, p.37]. When no reference standard exists: “*the accuracy of the proposed test itself will need to be demonstrated by direct from test to health outcomes evidence showing a health benefit resulting from use of the test, or by comparison against a suitable clinical utility standard*.” [1, p.38].

The ‘clinical utility standard’ is defined as: “*A special type of reference standard is the test that was used in the generation of direct from test to health outcomes evidence, which establishes the clinical utility of a test. This test (including the method of acquiring the sample, testing characteristics and interpretation of the results) is called the clinical utility standard*.” [1, p.38]

Outcomes: The identification and prioritisation of outcomes in the PICO is explicitly aligned to the nature of the test’s claim for improved patient health: “*The PICO to support claims of value of knowing must include outcomes that measure both the health-related claim (noninferiority/superiority) and the value of knowing claim. Outcomes for a value of knowing claim would include the types of options that become available to, or withdrawn from, an individual, or their family, as a consequence of information provided by a test. These options are not likely to be clinical (otherwise the claim would be for health outcomes), but may include the ability to make preparations, change behaviours or access support.*” [1, p.29].

MSAC cites guidance from GRADE to only include 7 key outcomes in summary of findings tables, which reflects the GRADE conclusion about the amount of information that decision-makers can balance at once [1, p.39].

MSAC notes the need to assess the availability of evidence when selecting outcomes: “*The harms and benefits of treatment or intervention following a test (for both test positive and test negative populations) are relevant outcomes. Direct from test to health outcomes evidence provides relevant harms and benefits (as long as the outcomes reported are relevant). If an investigative technology requires a linked evidence approach to demonstrate clinical utility, test performance and change in management are required outcomes*.” [1, p.40].

Types of outcome are listed, organised as test accuracy outcomes (including concordance with the clinical utility standard, diagnostic accuracy measures, and longitudinal accuracy, change in management outcomes (diagnostic thinking and subsequent testing), “*changes in preventive or therapeutic strategies*”, adherence to treatment, and “*outcomes related to referral patterns and/or frequency and timing of follow–up*”) and patient health outcomes. Examples of additional ‘value of knowing’ outcomes are also given “*where no change in patient management occurs as a result of the test information*” [1, p.41].

2. Review and post–review processes: we looked for information on:

- *What approach is used to develop evidence into guidance? e.g. by use of a committee to consider evidence reviews alongside any consultation processes*
- *How guidance is disseminated*
- *How patients or public, test developers, and/or experts are involved in the review process.*

**Process for involving Stakeholders:**

The *Process Framework* clearly summarises how major stakeholders are involved throughout the assessment process [2, p.15–16]. Consumer representatives and clinical experts are involved as members of MSAC and its two subcommittees [9]: “*MSAC is an independent expert committee comprising individuals from the fields of clinical medicine, health economics and consumer matters*.” [1, p.11].

Any consumer/patient can provide feedback at each major stage of the HTA process, mainly by accessing the completed Application Form and PICO Confirmation documents that MSAC makes available on its website. Targeted consultation of the initial application document is also undertaken. In addition to the input of sitting consumer committee members, MSAC also considers broader public feedback during its appraisal of the assessment (HTA) report [2, p.15–16].

Test developers (as applicants) are considered as major stakeholders during the triage process of application forms, as well as during development of the PICO**.** MSAC also considers its feedback (if they are the applicant) on the ESC report during its appraisal of the assessment report.

The majority of current MSAC members are clinical experts from a wide range of clinical specialties [9], and so are capable of appraising all the information generated through the HTA process. In addition, relevant medical professionals are involved as major stakeholders during triage of application forms, PICO confirmation documents and assessment reports.

**Processes for developing evidence into guidance and disseminating guidance:**

Assessment Reports for MSAC undergo a detailed peer review process during the ‘Assessment Stage’, during which the report is examined by the HTA group and the Evaluation Sub–Committee (ESC) for quality, validity and relevance [2, p.15–16]. The ESC produces a report which is passed to MSAC for consideration alongside the assessment report itself, the HTA group’s independent commentary on the report, feedback from the applicant on the assessment report, commentary and ESC report, and consultation feedback from any other relevant parties. Views are sought from individual MSAC members as well as external specialists (such as relevant professional bodies or specialists) and appropriate consumer bodies. Applicants are given the opportunity to reply to these views from the consultation feedback.

Applicants can choose to submit the assessment report ‘Context and Clinical Evaluation’ sections to ESC/MSAC first, so as to benefit from feedback on clinical evidence and the proposed structure of economic model at an earlier stage. In these cases, MSAC considers the evidence twice.

MSAC publishes its advice to the Minister for Health on its website as a Public Summary Document [2, p.15-16].

3. Recommended methods for performing a clinical effectiveness review of a diagnostic test: we looked for guidance on:

- *whether the HTA involves collection of evidence for review of clinical effectiveness;*
- *who collects the information and how this is funded;*
- *which evidence should be sought for tests, and using which search methods;*
- *methods to appraise the quality of evidence, and how to incorporate these into conclusions;*
- *summarising and synthesizing evidence*
- *methods to judge the relative strength and importance of the evidence? (e.g. GRADE)*

Overview of guidance on methods for Health Technology Assessments of tests:

MSAC has produced one methods guide for undertaking HTA of all healthcare technologies within its remit [1]. Methods for reviewing tests (as opposed to therapeutic devices) are provided in each key section of the MSAC *Guidelines*, with a dedicated substantive clinical evaluation section focussing solely on methods for the ‘*Assessment of investigative technologies*’ [1, chapter 2B]. Two appendices also focus exclusively on tests (‘*Test accuracy* measures’ [1, Appendix 7, p.271] and their link to specific therapeutic interventions ‘*Codependent technologies*’ [1, Appendix 8, p.282]).

A key process decision leading up to MSAC is whether to assess applications using an HTA framework or not. The decision to require an HTA framework is based on the type and materiality of change to the medical service. Full HTAs are only required when the clinical change is a:

- “*New (or change to existing) therapeutic or investigative service that is a significant variation to existing clinical practice or is materially changing how a current service is clinically delivered. May be accompanied by a proposal for new funding or a change to the existing funding mechanism/schedule fee*”
- “*New (or change to existing) specific consultation item where health outcomes can be measured*”
- “*New (or change to existing) co-dependent investigative service (bio-marker) with a drug*”
- “*Change reflecting who delivers a service involves systematic review of clinical evidence, alongside an assessment of translation issues and health economics analysis*.” [2, Figure 9, p.24].

MSAC notes that most applications require a full HTA. Specifically for tests, an ‘exemplar/facilitated assessment’ may be used which is an alternative approach to allow a shortened pathway of evaluation, mainly (though not solely) of relevance to genetic tests: “*For some genetic tests involving the assessment of multiple, related genetic variants, a simplified approach for some of the less common genetic variants may be reasonable*” [1, p.48]. Detailed criteria and processes for justifying and following a shortened HTA are provided in the MSAC *Guidelines* [1, p.49–53].

Within the HTA pathway, assessment reports consist of the systematic collection of evidence, comparative clinical effectiveness and safety review, cost–effectiveness analysis, and “an estimate of the utilisation of a technology, and the financial impact for the Australian Government or funder” [1, p.13]. Additional aspects are also systematically considered, most notably equity [1, TG29], value of knowing [1, TG28], presence of effective alternatives [1, TG29.7], and organisational issues including efficiency of health delivery, ethical concerns and social aspects [1, TG 29].

Methods for reviewing clinical evidence of diagnostic tests:

Methods follow the commonly accepted methods for conducting rigorous systematic reviews, with detailed specific tailoring to the evaluation of medical tests.

All the methods involved in the clinical effectiveness review, from decisions of which evidence to search for and collect to the analysis and synthesis of information, should be informed by the test’s clinical claim for improved effectiveness or not. An ‘assessment framework’ is provided to assist reviewers to articulate the specifics of the test’s claim in a systematic and transparent way. This framework is a structure for describing “*each step from testing to health outcomes*” [1, p.74]. It is mandatory for tests, and MSAC alerts reviewers that more than one may be needed if the test is used for multiple purposes. The assessment framework is adapted from the USPSTF analytic framework (USPSTF 2015 procedure manual), and similarly consists of a flow–chart graphic which should be populated with the review topic populations, intervention steps, and outcomes (in line with the PICO) as well as actions or inferences that link each box. The role of the study test (add–on, replacement, triage) is considered fundamental in constructing the detail of the claim, e.g. “*An add-on test will have additional costs, which must be justified by either superior health outcomes or strong logic for value of knowing, while a replacement test may be able to use a truncated framework*.” [1, p.74].

MSAC provides detailed guidance on what information the initial framework should contain as a minimum [1, p.75–6], and a key method is that each link between steps (denoted by arrows) must be accompanied by a question, which guides the evidence needed.

To assist this task, MSAC provides detailed example frameworks with associated assessment questions, which also include articulations of when it is appropriate to ‘truncate’ the framework for a claim of noninferiority (no difference in health outcomes), for example when comparing tests with similar accuracy [1, p.82]: “*A complete assessment framework is required for a claim of superior health outcomes, but the assessment framework may be truncated or adapted for claims of noninferiority or for value of knowing*” [1, p.74].

Further detailed guidance for constructing assessment frameworks (and related questions) for different test uses and comparisons are provided in Appendix 1, such as for: triage testing, a test replacing multiple tests (‘more definitive testing’), monitoring, multifactorial algorithms and machine learning/artificial intelligence tests, and screening tests [1, p.232–240]. Detailed guidance is also provided for adapting the framework to ‘value of knowing’ claims 1, p.85–87].

Importantly, the framework ties together the clinical effectiveness and cost–effectiveness analyses: “*The assessment framework describes the evidence required to verify the clinical claim, and to support the economic approach*” [1, p.74].

**What evidence should be collected?**

Direct evidence is preferred, and if present and sufficient no other evidence is needed. Direct evidence is defined as studies that evaluate all steps between performing the test across an eligible population and measuring consequential health outcomes which vary according to the test results:

“*A study that provides information on the categorisation of patients and their subsequent health outcomes is considered to provide ‘direct from test to health outcomes evidence’ only if 1) the treatment (and therefore health outcomes) was influenced by the results of the test, or 2) the study provides sufficient test result and treatment arms to make it clear whether following results of the test affected health outcomes*.” [1, p.91–92].

MSAC notes that direct evidence is rarely available and when present, studies often suffer from inadequacies, particularly transferability/generalisability to the target population, and underpowering to detect a difference in health outcomes [1, p.94]. Highlighting that most reviews will require a ‘linked evidence approach’, MSAC provides several chapters of detailed guidance on which indirect evidence should be sought for undertaking this method [1, TG11–13, p.99–129].

Evidence should be sought to answer all relevant assessment questions, which are organised into 3 incremental linkages:

- Evidence of test accuracy [1, TG11]:

“*In the absence of high quality direct from test to health outcomes evidence, an assessment will take a linked evidence approach. One key uncertainty with this approach is whether patients are appropriately categorised by the test (i.e. test accuracy). This information is needed so that the flow-on effects of test categorisation on subsequent evidence linkages can be determined (i.e. how the proposed test would change patient management and its likely impact on patient health outcomes).*” [1, p.99].

- Evidence of change in management [1, TG12]:

“*Change in management involves several sequential steps. Evidence may represent how a test result is interpreted (diagnostic thinking), what recommendations are made, and what is adopted by patients (i.e. the actual change in management).*” [1, p.117)

- Evidence of ‘impact of change in management on outcomes’ [1, TG13]:

“*Therapeutic effectiveness evidence, as the final step of the linked evidence approach, includes an estimate of the impact of all the management decisions made as a consequence of using the proposed test in the place of standard practice*.” [1, p.123].

MSAC describes a clear stepped approach to undertaking this phase of the assessment [1, p.88–89].

The adequacy with which these different evidence types can be linked forms a key MSAC method:

“*Discuss the consistency and transitivity of the evidence across the framework: Direct from test to health outcomes evidence is preferred to linked evidence. Uncertainty increases with the number of steps between the decision to test and the final health outcomes.^20^ As with any linked evidence approach, one source of uncertainty is the transitivity of the evidence across each step*.” [1, p.89].

Subsequent steps include examining the applicability of evidence to the target population and setting, social/ethical/legal/organisational issues associated with implementing the study test, and summarising results.

The use of expert opinion “*to supplement or support the observed data from randomised trials or nonrandomised studies*” [1, p.284] is suggested as a method to obtain data in the case of an ‘information gap’, with guidance focussing on how to report the use of expert opinion in a special appendix [1, Appendix 9, p. 284–287].

**Searching for** **evidence:**

Commonly accepted systematic search methods are described, with notable additional considerations for tests including the need for multiple searches, regardless of whether a linked evidence approach is required [1, Appendix 2].

Test–related search strategies should include test terms (study and comparator tests) as well as the target condition, to identify direct evidence and “*if taking a linked evidence approach, the test-related evidence up to change in management*.” [1, p.244].

MSAC specifies a separate treatment–related search is required for studies evaluating the impact of change in treatment on health outcomes, with detailed guidance to consider a separate search for *each* *change in management* occurring as result of the test [1, p.245–246]. Further, rather than the usual systematic approach to searching, MSAC recommends this is an iterative approach: “*because it may not be apparent that the identified evidence is suitable until an assessment of the evidence for applicability and generalisability has been performed. Following this assessment, should the evidence be rejected, further searches may be required. It is not expected that these searches are performed systematically, rather, that targeted searches are performed to try and identify the highest level, or best, evidence that addresses the impact of the change in management.*” [1, 125].

MSAC also notes that additional searching may be required to supplement direct evidence that does not compare the study test against the main comparator, in order to identify clinical utility data for that comparator.

A third search strategy may be required to systematically identify evidence on safety outcomes [1, TG14].

Lastly, guidance is also provided for searching for ‘value of knowing’ claims, noting “*methods for assessing the value of knowing are still in development*.” [1, p.246].

Caution is advised on using search filters, particularly for RCT and systematic review study types with MSAC emphasising these “*are unlikely to provide information required to undertake a linked approach.*” [1, p.244]. MSAC also cautions not to use test performance filters as these studies will not capture data measuring the impact of change in management studies [1, p.245].

In the case that insufficient evidence is identified for any part of the assessment framework, MSAC suggests reviewers consider seeking expert opinion and consumer–input [1, p.247].

**How should quality be assessed?**

Detailed considerations for assessing risk of bias are provided in Appendix 3, which provides a summary of the general principles of methodological quality and bias:

“*The methods for assessing therapeutic technologies are well established, and therefore quite stable. However, methods for assessing investigative technologies are less established, and new guidance is provided in the appendixes [sic] on using risk of bias tools to assess a range of tests with different uses, and how to adapt the GRADE approach for different linked evidence components.*” [1, p.48]

A table of validated tools to use for each study design is provided, with test–specific tools recommended for test accuracy studies (QUADAS–2) and prognostic studies (QUIPS) [1, p.251–2].

The applicability of studies to the review questions should be assessed for all evidence types, including for direct evidence where studies often suffer from poor generalisability [1, p.95]. Detailed discussion is given to the ways in which the generalisability of various study designs may falter, covering study population, study test, clinical decision–making, treatment and management.

**How should evidence be synthesised?**

Methods for summarising direct evidence (where complete) should be summarised as for therapeutic interventions [1, p.93]. Again the applicability/generalisability of studies is a central theme of guidance, with strong notes of caution that indirect comparisons of multiple direct evidence studies “*should be avoided, where possible*” due to ‘transitivity issues’ for multiple features of the comparison, specifically: population, prevalence, test thresholds and positivity rates, clinical practice decisions and treatment options [1, p.94].

Key guidance is given to when direct evidence does not completely address the PICO comparison:

A ‘clinical utility standard’ (in previous guidance termed the ‘evidentiary standard’) should be compared to the same reference standard as the proposed test. Specifically, this involves a comparison of concordance in diagnostic decision-making (rather than just accuracy) when the proposed test is being compared against the clinical utility standard (the test used in the direct evidence study):

“*If direct from test to health outcomes evidence is available, and the proposed test is not the clinical utility standard, the most important comparison is of the proposed test vs the clinical utility standard. The comparison reports the nature of discordance and the implications*.” [1, Figure 16 note c, p.101].

“*Equivalence of the proposed test and the clinical utility standard provide confidence that the proposed test will result in the same health outcomes as observed in the study that used the clinical utility standard*.” [1, p.101].

However, we are unclear whether the ‘clinical utility standard’ is only relevant to the evaluation of codependent investigative technologies:

“*A special type of reference standard is the test that was used in the generation of direct from test to health outcomes evidence, which establishes the clinical utility of a test. This test (including the method of acquiring the sample, testing characteristics and interpretation of the results) is called the clinical utility standard*.” [1, p.38]

MSAC provides detailed guidance for linked evidence approaches, with a chapter dedicated to each major link in the evidence pathway.

Link 1: test accuracy

Standard key concepts and methods for estimating and synthesising test accuracy are provided [1, TG11 and Appendix 7] with the following notable additions:

- Evidence of comparative accuracy is required, although whether this should be achieved through direct or indirect comparisons is not addressed [1, p.101];
- Reference standard and imperfect reference standard considerations [1, p.102–106];
- importance of discussing the nature of discordant cases (TP and FN):

“*A key uncertainty in the linked evidence approach arises when the proposed test categorises patients differently to the comparator (e.g. results in more or fewer diagnoses), as it is unclear whether these differences represent a change in the spectrum of the disease being identified, or some other systematic reason for differences between the tests. Where there is a change in the types of patients categorised as test positive and test negative, there is less certainty that the subsequent steps in a linked evidence approach (change in management and health outcomes) would be applicable if these steps were informed by a standard that categorises patients differently*.” [1, p.100].

- MSAC makes a distinction between ‘clinical’ and ‘nonclinical’ target conditions:

“*Use of a clinical versus a nonclinical reference standard when evaluating test accuracy: In some circumstances (such as biochemical, cytogenetic and molecular genetic testing), it is important to distinguish between how accurate the test is in detecting a biomarker and how accurate it is in detecting the clinical disorder or outcome of interest. If good quality and applicable estimates of accuracy against a valid clinical reference standard are available, test accuracy against a nonclinical reference standard may not needed*.” [1, p.104].

Further, “*In the absence of a clinical reference standard, the clinical accuracy of a test depends on both the ability of the test to detect the biomarker compared to the nonclinical reference standard as well as the strength of the biological plausibility linking the surrogate measure (test results) with the clinical condition of interest*.” [1, p.105].

- MSAC recommends assessing publication bias [1, Appendix 4].
- Special considerations for prognostic and predictive tests, companion diagnostic tests, and genetic cascade testing.

Link 2: Change in management:

MSAC provides a clear philosophy in accordance with the assessment framework: “*An impact of a test on health outcomes can only be achieved if the interpretation of the test results leads to a change in the management for a patient*.” [1, p.116].

“*Change in management involves several sequential steps. Evidence may represent how a test result is interpreted (diagnostic thinking), what recommendations are made, and what is adopted by patients (i.e. the actual change in management)*.” [1, p.117].

The broad method is to: “*Present the evidence for change in management in the same way as for a therapeutic health technology*” [1, p.116].

Detailed explanation is given to the multiple factors that determine whether a test can change clinical management, including availability of treatments, differences in test results (accuracy), whether results change ‘diagnostic thinking’, whether patients are willing to take the treatments and patient adherence [1, p.117].

MSAC also includes changes to timing of treatment (early vs late intervention) in this domain.

Caution is noted for using historical control and before–after designs (the latter of which should not be used for replacement or triage tests at all), with guidance that these studies are subject to bias [1, p.119].

Change in management evidence should be summarised using the same approach as for therapeutic interventions (including meta–analysis when appropriate), and in addition providing a discussion of the reasons for variation in clinical management of patients with the same test results. Some guidance for considering and incorporating the applicability of evidence is provided [1, TG 12.5].

Link 3: Health outcomes

The last link in the chain of evidence should seek evidence of therapeutic effectiveness: “*In general, therapeutic effectiveness evidence should attempt to derive the highest quality evidence for the incremental difference in outcomes associated with treatment decisions informed by the proposed test versus treatment decisions informed by the comparator test*.” [1, p.123–4].

“*The principles for presenting health outcome gains evidence are similar to those for presenting clinical study evidence for a therapeutic health technology*” [1, p.129].

Consideration is given to the information that various study types can provide:

“*The following general guidelines may assist in determining the types of studies that may be useful:*

- *Comparative studies are useful to explore the impact of changing from one management strategy to another.*
- *A relative treatment effect is not useful to describe the differences between treatments that are prescribed for different test populations (e.g. positive and negative biomarker status).*
- *Observational studies are useful for determining the natural history of the disease.*
- *Studies comparing the outcomes of the same treatment by biomarker status may also be useful in identifying whether a patient’s biomarker status has a prognostic effect. Understanding whether the biomarker is prognostic or not may inform whether evidence from unselected populations receiving treatment A can be generalised to test-selected populations using treatment A.*
- *Studies reporting on subgroup analyses defined by population characteristics or biomarker status may be useful for determining the applicability of the evidence to the target population.*” [1, p.125].

A notable departure from therapeutic technologies is the need to carefully consider the “*generalisability of the evidence across differently selected populations*” [1, p.124]. MSAC notes that generalisability in this section of linked evidence tends to be the most problematic of all linkages, as: “*if the test is relatively new, there is unlikely to be evidence for the outcomes of patients allocated to treatments according to results of the proposed test. Therefore, health outcomes evidence for the treatments identified in the change in management section may not be generalisable to the population receiving that treatment following the use of the proposed test in practice*” [1, p.125].

Applicability should be assessed as for a therapeutic intervention [1, p.128].

Detailed guidance is provided on the components to consider for this evidence, all of which are related to whether treatments exist and are effective, and the importance/consequences of test misclassifications. The safety of downstream effects should be included in this section [1, p.129].

MSAC also underlines the importance of carefully examining the potential for the new test to change the spectrum of patients receiving treatment; if for example the study test is more sensitive, then previously missed patients will receive treatment at potentially earlier stages of disease: “*A comparison of early versus late treatment may be informative*” [1, p.127].

Evidence for impact to health outcomes should be summarised using the same approach as for therapeutic interventions, with these additions [1, p.129]:

- Provide an “*assessment of the outcomes relating to the change in management (e.g. if a test results in 20% of patients receiving Treatment A instead of Treatment B, a comparison of Treatment A versus Treatment B is appropriate)*.”
- Description of the assumptions and justifications of generalisability.

MSAC includes specific guidance for incorporating evidence of safety [1, TG14], consisting of 3 key elements for tests:

1. Direct health impacts, such as adverse events from undergoing the test itself [1, p.131–132].
2. Downstream harms following management decisions of the test, which should be assessed using similar methods as for therapeutic technologies.
3. Longer term or rarer safety events unlikely to be captured in clinical studies [1, p.130].

“*[A] narrative synthesis that discusses the entire safety profile may be informative, particularly in cases where there is a trade–off (e.g. the proposed test has an inferior safety profile compared with the comparator, but the test results in improved safety outcomes for treatment*” [1, p.130].

For tests with additional claims that are considered by MSAC not to be health–related, guidance on how to summarise ‘value of knowing’ evidence recommends describing key benefits and harms in tabular form, comparing the effect in patients receiving the study test versus comparator test [1, p.223]. Suggestions are offered on how ‘value of knowing’ evidence can be incorporated into the health economic evaluation particularly through use of a cost-consequences analysis [1, p224].

**How should the strength of the body of evidence be determined?**

Grading the body of evidence is considered in Appendix 4 [1, p.258–262], where MSAC recommends using the GRADE (*Grading of Recommendations, Assessment, Development and Evaluations*) approach. Broadly, ratings should be undertaken for the overall body of evidence, for each outcome: “*An overall assessment of the quality of the evidence for each outcome is necessary to indicate the certainty that MSAC may have that the evidence represents the ‘true’ effect of the health technology.*” [1, p.258].

Specific considerations for tests include:

Recommended use of the standard GRADE approach for direct evidence, “*with consideration of how additional applicability issues are addressed*”. This includes down–rating any outcome measures that are not “*directly patient relevant*” [1, p.260].

For linked indirect evidence, recommendations to consider the quality of links by adapting the GRADE process: “*Elements that could affect the quality of the evidence could include variability in characteristics of the test population, differing test result definitions that lead to a change in management and differing options for treatment based on the same test result*.” [1, p.260].

For change in management and treatment efficacy, the standard GRADE approach is again recommended, however outcomes should not be down–rated for indirectness with a caveat: “*The outcomes may be rated down for indirectness due to other reasons (e.g. indirectness of outcome if a change in diagnosis is reported, rather than a change in management; if outcomes reported are management recommendations, not management received; or indirectness of population for therapeutic efficacy, if the spectrum of patients treated does not match those who are likely to be treated based on test results)*.” [1, p.261].

MSAC notes that for rating accuracy studies: “*Using the GRADE approach for questions regarding the diagnostic accuracy of a test are well established*” [1, p.261]. However it recommends that rather than the outcomes of true/false negatives/positives: “*For the purpose of population-based funding decisions, it is suggested that the more relevant outcome measures (which are less susceptible to pre-test probability differences) are sensitivity and specificity.*” [1, p.261].

In addition: “*Assessment should consider both the quality of evidence as it relates to test accuracy, as well as the proportion of patients with unevaluable results or inconclusive results (who may need to have a second sample retrieved).*”

MSAC also provides guidance for how to adapt the GRADE approach for predictive tests, which must be evaluated using longitudinal (rather than cross–sectional) accuracy studies [1, p.262].

**Overall interpretation of evidence for tests:**

A chapter of the MSAC *Guidelines* is devoted to guidance for summarising the overall body of evidence for an investigative technology [1, TG16]:

**“***Summarising the evidence base for a test must account for the quality and strength of the evidence for each individual component, as well as an overall assessment of the effect of the proposed test on health (or other) outcomes.*” [1, p.148].

Guidance is provided for structuring summaries of evidence, and underlines the importance of conveying key uncertainties identified “*for each evidentiary step*”.

Linked evidence should be summarised narratively: “*The conclusion regarding the clinical utility of the investigative technology should be a simple and unequivocal statement about the superiority, inferiority or noninferiority of the test to the comparator and/or reference standard that is supported by evidence provided in the submission*” [1, p.148].

MSAC provides useful suggestions for producing an informative narrative, including explaining the summative benefits and harms for a hypothetical population from the point of undergoing testing: “*and follow the population through to the range of different outcomes, incorporating change in management, the proportion treated appropriately versus inappropriately (based on accuracy of test results), and the patient-relevant health outcomes. This creates a comparative effectiveness model, which can then be expanded on to form the basis of a cost-effectiveness model in the economic evaluation. The model, or narrative synthesis, of clinical utility should capture the trade-off inherent in testing and subsequent decisions. It should also identify crucial areas of uncertainty in the existing data where more primary data collection is required*” [1, p.148].

4. Recommended methods for evaluating the cost–effectiveness of diagnostic interventions: we looked for guidance on:

- *whether the HTA should involve any assessment of cost–effectiveness, and if so which methods are recommended (e.g. cost–utility, ‘cost–effectiveness’, cost–minimisation, budget impact analysis);*
- *how evidence should be incorporated into health economic models;*
- *how the clinical effectiveness review and cost–effectiveness study should link together (e.g. the extent to which clinical effectiveness review results are used to inform economic models);*
- *methods for carrying out linked evidence modelling, where this is different to health economic modelling*

**Methods for conducting Health Economics assessments**

Health economics assessments constitute a major part of HTA, and guidance is provided in Section 3 of the MSAC *Guidelines* [1, p.154–205]. An estimate of use and financial implications (budget impact) is also always required and described in Section 4 [1, p.206–219].

Separate guidance is provided for a cost-effectiveness analysis (CEA) (including cost-utility analysis, CUA) [1, Section 3A], and for a cost-minimisation approach [1, Section 3B]. CEA is used when the technology is claimed to be superior to the comparator; cost-minimisation when the technology has been demonstrated to be non-inferior to its main comparator(s) in terms of both effectiveness and safety.

The methods which are described, although detailed, cover the general approaches to conducting a modelled estimate of cost-effectiveness consistent with any standard text-book. There is a reference case for economic evaluations [1, Table 13, p.155-156]. The assessment question (including the comparator) should be "*as defined in the PICO confirmation*”. A preference for cost-utility as the measure of cost-effectiveness is indicated. The perspective is the personal health of the person receiving the intervention for benefits, and the health care system for costs. Estimates of effectiveness should be “*derived from the systematic review conducted in Section 2, translated as necessary*”. The discount rate for costs and benefits is 5%.

For CEA “*MSAC prefers that the economic evaluation is based on results from direct randomised trials, with adjustments or additions to the trial data as required to account for differences in the population and setting, timeframe of analysis or outcomes of interest*.” [1, p154]. Any adjustments required to accommodate different types of evidence should be presented in a stepped manner to see what the effects of these adjustments are.

Special considerations for issues for assessing the cost-effectiveness of tests are mentioned:

- Assessment question for economic evaluation should include diagnostic decisions and outcomes [1, p.157], as illustrated in Figure 25 [1, p.159].
- Concerning perspective, both benefits and harms directly related to the service (e.g. an adverse event due to exposure to an imaging contrast agent) and those indirectly related to the service (such as those that arise from subsequent changes in treatment) should be included [1, p160].
- Concerning valuation of outcome, personal utility arising from knowledge of the test result (such as those that claim to assist with reproductive planning), can be included [1, p161].
- For tests the model structure may need to account for prevalence of disease (or risk stratification for a prognostic technology), test accuracy (including cost and health outcome implications for patients who receive a false result or those in whom testing fails), change in management, and the effect of change in management, where relevant [1, p164; and illustrated in Figure 26 p.165].
- For evaluations in which it is reasonable to model multiple distinct populations (such as where a test identifies multiple distinct diseases with differing treatments and prognoses), the model structuring process should be performed for each distinct population [1, p164].
- All patients eligible for the test should be included in the model, not just those that the test aims to identify [1, p.171].
- The list of model parameters should include test accuracy and changes in clinical management [1, p.174].
- In model validation, separate model traces for patients who do and do not have an appropriate change in management should be performed, to show the dilution effect associated with tests, whereby the test may only affect management in a subset of patients tested [1, p187].
- Concerning “stepping” the model, MSAC advises to consider sequentially incorporating evidence from each of the linkages. For example, first present costs and outcomes associated with test use based on test analytical data, then add change in management information, clinical outcomes and, finally, translated (e.g. extrapolated and/or transformed) health outcomes [1, p.188 with illustration in Table 17, p.192-193].
- In taking a cost minimisation approach, it would generally be sufficient to cost the health technologies in each arm to the point of diagnosis. However MSAC cautions that it is difficult to justify a cost-minimisation approach assuming final health outcomes were equivalent if the analytical test outcomes/diagnostic outcomes were not also equivalent [1, p.204].

There is a strong steer that the model should be shaped by the clinical claims in order to demonstrate what the cost-effectiveness of a technology meeting those claims is: “*The model structure should capture all relevant and important health states or clinical events along the disease or condition pathway, and should be consistent with the treatment and disease or condition algorithms created in response to Technical Guidance 2*” [1, p.164].

Given that evidence linkage is likely to be the most common way in which clinical effectiveness is demonstrated, this suggests that evidence linkage must also be intrinsic to the economic model. This is illustrated by the advice on “stepping” which suggests sequentially incorporating evidence from each of the linkages. “*For example, first present costs and outcomes associated with test use based on test analytical data, then add change in management information, clinical outcomes and, finally, translated (e.g. extrapolated and/or transformed) health outcomes*…” [1, p.188 with illustration Table 17, p.192-193].

In the MSAC *Guidelines*, “evidence linkage” refers to the approach to demonstrating clinical effectiveness by bringing together evidence on the effect of tests on different parts of the clinical pathway. It is “*When evidence from studies of test accuracy is linked to evidence of change in management and evidence of treatment effectiveness to derive an estimate of the clinical utility of the test*”. [1, p.17]. It is separate from the process of economic modelling, although the modelling is informed by the evidence linkage arguments.

Irrespective of whether the evidence of effectiveness is direct or indirect (as in the case of evidence-linkage), the reference case indicates that the evidence should be systematically presented [1, p.156]. Translation of the evidence may also be required [1, p.172-3].

## Special features noted of the MSAC methods and processes:

- Rigorously developed process framework [2] that incorporates tailoring for HTAs of tests.
- The health–related claims of testing underpin all elements of the HTA approach, and a structure is provided to assemble the claim in a standardised manner, the ‘assessment framework’.
- The review of clinical evidence and the cost–effectiveness analysis are explicitly linked by the claims of the test.
- Specific and extended information on what is meant by a linked evidence approach (TG11–13).
- Separating non-inferiority and superiority of accuracy, with some detailed guidance e.g. Fig 9 [1, p.83] and Appendix 5 [1, p.267].
- Specific considerations of other test types throughout the MSAC *Guidelines*, including screening, monitoring, prognosis, predictive, genetic and artificial intelligence/machine learning tests.
- An additional special chapter (Technical Guidance 15 Special cases) is included for particular types of tests, discussing the following key considerations:
  - “*Screening can encompass broad or narrow populations. Considerations relevant to the assessment of universal screening programs, targeted screening, predisposition testing and cascade testing will differ* (TG 15.1).
  - *Tests used to determine prognosis may have value relating to improved health outcomes if they inform change in management, or only have benefits that fall within the concept of value of knowing. An assessment of a prognostic test reports how well a test can differentiate future health events compared with current prognostic (often clinical) tests, as well as how this will impact clinical management* (TG 15.2).
  - *Predictive tests are used to determine how well a patient would respond to a treatment. Typically, these tests identify a biomarker that inform the eligibility for a treatment, and is most commonly applied in a co-dependent context. A critical concept within predictive testing is the clinical utility standard, and an assessment includes a robust comparison of the proposed test against the clinical utility standard* (TG 15.3).
  - *The assessment of a test used for monitoring is similar to the assessment of tests for other purposes, using either a direct or linked evidence framework. However, additional aspects of the test, such as its responsiveness to changes in response to an intervention (signal to noise ratio), the detectability of long-term change, and the ease of use and interpretation of the test, should be discussed* (TG 15.4).
  - *An assessment of a multifactorial algorithm involves the presentation of the biological plausibility, the characteristics of the training and validation sets (for establishing applicability), and additional results to ensure that algorithms (particularly dynamic algorithms) are not subject to bias when used in the Australian population* (TG 15.5).
  - *A codependent submission is required when the Minister for Health requires advice from 2 different expert advisory committees because listing of the codependent technologies involve 2 separate reimbursement schemes* (TG 15.6).” [1, p.134].
- Clarification of the specific circumstances in which expert opinion may be useful [1, Appendix 9].
- Specific consideration of impacts that are not health–related: Equity (TG29), Value of knowing (TG28), presence of effective alternatives (TG29.7), organisational issues including efficiency of health delivery, ethical concerns, social aspects and legal concerns (TG 29). Environmental aspects are also mentioned (e.g. reduction in emissions) [1, p.230].

**Questions to ask MSAC:**

1. **Is the content of our summary above a fair and accurate reflection of your organisation’s processes and methods for undertaking HTAs of diagnostic tests?**

Yes.

1. **Are there any publicly available documents we have missed that outline your processes for undertaking HTAs of tests?** We would like to provide a summary of organisations’ procedures for: topic referrals, pre–HTA scoping/topic development phase, involvement of stakeholders, post–review development of HTA into guidance**.**

I have already provided the public consultation update (which is applicable to MSAC-related HTA more broadly than of tests). The MSAC website, which you have already extracted documents from, contains all the external facing information on these topics. I do not think there is anything missing. However your approach to the question reflects the dominance of NICE’s approach to HTA for your list of procedures, so to translate:

- MSAC receives applications for health technologies and medical services from applicants, which is the closest parallel to NICE’s “topic referrals”.
- The pre-assessment stage of the MSAC process usually comprises a suitability/triage step and a PICO Confirmation step, which is the closest parallel to NICE’s “pre-HTA scoping/topic development phase”.
- MSAC’s public consultation approach is the closest parallel to NICE’s “involvement of stakeholders”.
- MSAC publishes its advice, and the basis of and rationale for this advice, in the form of Public Summary Documents (PSDs) for each of its considerations, see [MSAC - Application Page](http://www.msac.gov.au/internet/msac/publishing.nsf/Content/application-page), which is the closest parallel to NICE’s “post-review development of HTA into guidance”. Note that, in the same way that NHS England implements NICE guidance documents, other entities consider and implement MSAC advice.

1. **Are test developers, patients and experts involved in any part of the HTA process?** We have noted that consumers/public, clinical experts and applicants are involved in various stages of producing MSAC assessments. Are test developers involved at any stage, when they are not the applicant?

I confirm that the public, comprising consumers, patients, clinical experts, competing providers etc can all provide input via the public consultation process, and that this will be considered by MSAC alongside the other documents generated as a result of accepting an application for consideration. So “test developers” could take this opportunity. However, note that the developer of a test would generally become the applicant for the test. Australian test developers are usually either commercial enterprises developing test kits which are approved by Australia’s medical device regulatory procedures, or pathology laboratories developing “in house” tests which are approved through another national pathology accreditation and quality assurance process and notified to Australia’s medical device regulatory agency. The MBS preference is to be agnostic of the test methodology when defining the scope of the funded test unless there is evidence of insufficient concordance (hence MSAC’s emphasis on concepts such as the clinical utility standard). It might also be relevant that MSAC and its sub-committees have members with consumer, pathology and imaging expertise, and this expertise is also available in the areas of the Australian Government Department of Health which supports MSAC.

1. **Are there any publicly available documents we have missed that outline the methods that should be used in an HTA of diagnostic tests?**

Not substantially. The previous standalone Clinical Utility Card Proforma for use in predisposition and associated cascade testing has been absorbed into the 2021 MSAC Guidelines. Two reports under the “Other Resources” option under the “Forms Templates & Guidelines” tab of the MSAC website (see [MSAC - Additional Resources](http://www.msac.gov.au/internet/msac/publishing.nsf/Content/Additional-Resources)) anticipate issues that arise for tumour-agnostic biomarker testing to determine eligibility for targeted treatment. This includes a focus on mismatch repair deficiency testing and checkpoint inhibitors as an example. These issues have not yet evolved to the point that they could be described as “methods that should be used in an HTA of diagnostic tests”. That work is ongoing.

1. We note in your Process Framework document that a 2–stage submission is allowed, in which MSAC considers clinical effectiveness evidence, before the cost–effectiveness analysis is conducted and submitted. **Is this still the case in light of your new methods guidance document?**

This remains an option. However, it is rarely taken up.

1. We are particularly interested in how test accuracy information is translated into conclusions about impact on patients outcomes. We note your 2021 Guidance has expanded its information to reviewers for performing a linked evidence approach. **Your methods imply to us you do not perform mathematical modelling to estimate the health impacts of diagnostic tests. Is this correct?**

No. Implicit (if not explicit) in the “linked evidence approach” is the need for mathematical modelling to link multiple sources of evidence to connect test accuracy information to conclusions about health outcomes. This is necessary to give a quantified estimate of the incremental health outcomes gained (or to support a claim of noninferiority). The mathematical modelling component of this definitely formalised in the modelling required to construct the associated economic evaluation which enables an assessment of the incremental costs against these incremental health outcomes.

1. We note that in certain circumstances you recommend comparing the proposed and comparator tests to a ‘clinical utility standard’, in order to estimate the degree of concordance in decision–making. **Could you please clarify whether this method should be limited to evaluations of codependent investigative technologies, or is it a key approach for any test whose comparator is known to be less accurate than the reference standard?**

This method is not limited to evaluations of codependent investigative technologies. Rather it is limited to where there are studies that directly measure health outcome consequences according to whether the proposed test is used or not. It provides a basis for judging whether other possible in-scope test options perform similarly enough to the test(s) included in these studies to be allowed to be used as alternatives. The approach does not rely on any judgement against a reference standard, indeed it can apply when a reference standard doesn’t exist.

## National Institute for Health and Care Excellence Diagnostics Assessment Programme (NICE DAP, England and Wales)

### Sources used for information

[1] Organisation website: <https://www.nice.org.uk/> [last accessed 18/06/21]

[2] ***Diagnostics Assessment Programme manual.*** NICE; December 2011. Downloaded 28^th^ April 2020.

[3] ***Medical technologies evaluation programme methods guide.*** NICE; August 2017. Downloaded 11^th^ June 2020.

[4] ***Interim Addendum to the Diagnostics Assessment Programme Manual*.** NICE webpage, available from <https://www.nice.org.uk/Media/Default/About/what-we-do/NICE-guidance/NICE-diagnostics-guidance/Diagnostics-interim-addendum-access-proposals.pdf> [last accessed 5^th^ July 2021]

[5] **Interim addendum to replace existing section 9, Guidance reviews, in DAP programme manual.** NICE webpage, available from <https://www.nice.org.uk/Media/Default/About/what-we-do/NICE-guidance/NICE-diagnostics-guidance/Diagnostics-interim-addendum-guidance-reviews.pdf> [last accessed 5^th^ July 2021]

### HTA setting

Health Technology Assessment (HTA) in England is led by the National Institute for Health and Care Excellence (NICE). HTA within NICE is undertaken by independently commissioned academic groups: Evidence Review Groups (ERGs). NICE is accountable to but independent from the UK Government and was created in 1999 to reduce variation in the availability and quality of NHS treatments and care; its role is *‘to improve outcomes for people using the NHS and other public health and social services’* [1]*.* NICE is accountable to, but remains independent from, the UK government. NICE guidance technically only covers England but decisions about how NICE guidance applies in other countries in the UK (Wales, Scotland and Northern Ireland) is made by the respective devolved administrations. These other UK countries are often involved and consulted on the development of NICE Guidance, and NICE has agreements to provide them with certain services and products.

1. Pre–evidence review processes for HTAs of tests: we looked for information on:

- *Where suggestions for which tests to review come from (referral source)*
- *Whether there are any entry criteria (e.g. regulatory conformity such as CE mark)*
- *Whether a formal scoping phase is recommended or mandated, and if so how the scope should be developed from the referred topic, and what the scope’s focus should be.*

### Processes for identifying diagnostic topics for HTA reports

The Medical Technology Evaluation Programme (MTEP) receives proposals for potential diagnostic topics (alongside other medical technologies) from clinicians and policy makers (including National Clinical Directors, Medical Royal Colleges, professional bodies, national expert bodies and national screening programmes) and industry. Industry are described as the *“main source”* of potential topics [2, pg. 30].

To be eligible for consideration a diagnostic technology must be a device (i.e. not elements of the clinical history and examination, questionnaires or structured interviews), the technology must have a CE mark, and the technology must not be being considered for or being used as part of national screening programmes [3, pg. 12]. Diagnostic technologies for any purpose (screening outside of national programmes, diagnosis, staging, risk stratification, and monitoring) are within the programme remit. Companion diagnostics for established treatments are also within the programme’s remit.

A topic briefing is prepared for eligible technologies incorporating “*input from expert advisors, patient and carer organisations if possible”* [3, pg. 10) and based on a set of predefined criteria [3, pg. 32]:

- measurable benefit to patients’ quality of life or life expectancy over currently available technologies
- healthcare system benefit (reduction in staff or facility resources)
- size of potentially impacted population
- impact of disease or condition on quality of life (severity)
- initial acquisition and running costs
- sustainability (e.g. reduction in energy requirement, reduction in waste)

Diagnostic technologies that are complex, for example where recommendations can only be made on the basis of cost-effectiveness analysis, where assessment requires consideration of multiple technologies or indications for testing, or for technologies that are likely to have more benefits but “*cost more than those in current use”*, are routed for consideration by the Diagnostics Assessment Programme (DAP) [3, pg. 8 and pg. 11). Diagnostic technologies that *“may offer similar health outcomes at less cost, or improved health outcomes at the same cost as current NHS practice”* are assessed by the MTEP [2, pg. 8],[3, pg. 11]

### Process for scoping HTA diagnostic topics

For technology appraisals undertaken by the MTEP, the scope is based on the sponsor’s case for adoption which may be supplemented with technical questions from the Medical Technology Assessment Committee (MTAC). The scope outlines the rationale for selection of the topic including any relevant equity considerations, a list of the professional and patient organisations who will be providing comments on the technology, and a list of the societies and organisations that will be invited to comment on the scope [3, pg. 13]. The scope will include details about the condition or clinical problem relevant to the technology (the *‘relevant population’)*, a description of the technology, its regulatory status and its claimed benefits, the comparator, and the effect of the technology on clinical and system outcomes.

For technology appraisals undertaken by the DAP, an extensive scoping process is undertaken, typically over a 14-week period, by the Specialist Committee specifically convened for assessment of the technology [2, pg. 35-41]. The committee comprises representatives from the topic sponsor(s), professional and lay members selected from applicants responding to adverts on the NICE website, registered stakeholders (including relevant clinical guideline groups, patient and carer organisations, and professional organisations including the Medical Royal Colleges [2, pg. 32-4] and the EAG.

Searches are undertaken within NICE by the DAP technical team and an initial draft scope is developed at a workshop attended by all specialist committee members prior to publication of a version for public consultation.

The scope outlines the proposed assessment of the technology (or multiple technologies) in terms of use in *specific* clinical circumstances delineated by Population, Intervention (test and comparators which should be considered in equivalent detail), Care pathway, Outcomes, and costs [2, pg. 36].

A key element of developing the scope is *“collecting information about outcomes (benefits and harms to the patient) through the entire care pathway, including post diagnosis’* [2, pg. 35]. This is referred to as *‘care pathway research”* [2, pg. 40]. Any direct or indirect health outcome experienced over a short or longer term resulting from the use of the test *“including information pertinent to relief of anxiety or personal planning; these include longer term outcomes in most cases”* may be considered [2, pg. 42]. There is no suggestion that outcomes might be prioritised, such as into those which are central to the case for the test and those which are not. However examples of where the scope may be shortened to include only the outcomes from the test and the test costs are provided, such as where accuracy of a test and its comparator is the same, or where the downstream outcomes from the intervention and comparator are expected to be the same. Similarly it is recognised that the assessment structure may be simplified if during scoping it is recognised that there are long term studies that enable determination of “*long-term outcomes without having to model this through intermediate outcomes”* [2, pg. 42].

2. Review and post–review processes: we looked for information on:

- *What approach is used to develop evidence into guidance? e.g. by use of a committee to consider evidence reviews alongside any consultation processes*
- *How guidance is disseminated*
- *How patients or public, test developers, and/or experts are involved in the review process.*

### Process for involving stakeholders

Patients and carer representatives are appointed as members of the specialist committees for both the MTEP and the DAP process. In this capacity they are involved at all stages from delineating the project scope through to development and dissemination of guidance. In addition, for the DAP, up to 20 places are open to members of the public and registered stakeholders for the Diagnostic Appraisal Committee meeting where a decision and recommendations are made about technologies. Test developers scope and produce their own evidence review and economic modelling for consideration by the specialist committee as part of the MTEP [3, pg. 11-18]. As part of the DAP, the technology sponsor and competing technology manufacturers are contacted at the initiation stage of the assessment process and invited to submit evidence to support the scoping process [2, pg. 11-15]. The EAG may request information from the sponsor and competing technology manufacturers, via the NICE topic lead, during the assessment process. Test developers can take part in consultation on the assessment report produced by the EAG and the draft and final guidance. They can attend the final Diagnostic Appraisal Committee meeting where decisions and recommendations are made but do not contribute to these decisions. Clinical experts are involved as stakeholders throughout the HTA process as summarised in sections Process for scoping HTA diagnostics topics, and Process for developing evidence into guidance.

### Processes for developing evidence into guidance and disseminating guidance

Process for developing evidence into guidance

For diagnostic technologies considered by the MTEP, the Medical Technologies Advisory Committee members themselves develop guidance, considering all evidence and contributions from expert advisers and patient and carer organisations. The exact nature of the final Guidance is determined by consideration of the potential benefits of the technology to patients and the health and social care system [3, pg. 21]. Draft guidance is made available on the NICE website for public consultation for a 4-week period [3, pg. 24].

For diagnostic technologies considered by the DAP, [2, pg. 46-51, and pg. 105-112] the EAG’s Diagnostic Assessment Report is considered as evidence at a Diagnostic Advisory Committee meeting attended by representatives from the EAG, the diagnostic technology committee convened specifically for the topic, sponsors, stakeholders, and the public. The key sources of evidence considered by the Diagnostic Advisory Committee in making recommendations for practice and research are diagnostic test accuracy, clinical effectiveness, and cost effectiveness [2, pg. 105-109]. Cost-effectiveness is the main aspect of the evidence mentioned in determining the types of recommendation [2, pg. 111] with greatest detail devoted to the criteria for making a decision about cost-effectiveness depending where the cost/QALY estimate is in relation to the decision-making thresholds of £20,000 and £30,000 per QALY. A framework for research recommendations is provided including the use of value of information analyses. The output of the Diagnostic Advisory Committee meeting is a consultation document (the Diagnostic Consultation Document) which forms the basis of the Diagnostics Guidance Document (DGD) following a period of public consultation.

Disseminating Guidance

For diagnostic technologies considered by the MTEP, final guidance and recommendations are published on the NICE website after the 4 week public consultation period. Recommendations may be supported by development and publication of implementation tools where relevant [3, pg. 8].

For diagnostic technologies considered by the DAP, all interim documents (Diagnostic Assessment Report, consultation comments and responses) and final outputs (the DGD, implementation support tools such as audit tools for use by the NHS and a lay explanation of the recommendations) are published on the NICE website. In situations where the Diagnostic Appraisal Committee does not recommend uptake of a diagnostic technology but accepts that a technology delivers measurable benefit and that the acquisition cost of the technology is a key driver of the ICER, sponsors may apply for an access proposal. If granted, the access proposal may involve an arrangement where the NHS can acquire the technology at a lower than normal acquisition price to facilitate uptake [4].

3. Recommended methods for performing a clinical effectiveness review of a diagnostic test: we looked for guidance on:

- *whether the HTA involves collection of evidence for review of clinical effectiveness;*
- *who collects the information and how this is funded;*
- *which evidence should be sought for tests, and using which search methods;*
- *methods to appraise the quality of evidence, and how to incorporate these into conclusions;*
- *summarising and synthesizing evidence*
- *methods to judge the relative strength and importance of the evidence? (e.g. GRADE)*

### Overview of guidance on methods for Health Technology Assessments of tests

NICE has a process and methods guide [3] which outlines the criteria considered for the purposes of routing medical devices (diagnostic and other types of technologies) to different elements of the MTEP programme as well as the methods guiding technology appraisal for less complex diagnostic technology assessments considered by the MTAC itself.

For complex diagnostic technologies routed to the DAP there is a separate manual which includes an introduction to diagnostic technologies (Part I), programme processes (Part II), and methods for undertaking HTA (Part III). PART III outlines methods for “*an assessment of the diagnostic test accuracy, clinical outcomes and cost effectiveness of the technologies… as well as modelling of patient outcomes, costs and cost-effectiveness”* [2, pg. 42]. The DAP manual was last updated in December 2011 [2].

### Methods for reviewing clinical evidence of diagnostic tests

For less complex diagnostic technologies undergoing assessment by the MTEP, evidence is collected from expert advisors and patient and carer organisations. The sponsor of the technology performs a review of published, unpublished, and on-going evidence in support of its submission and may also undertake a review of existing health economic evaluations rather than undertaking a de novo modelling exercise [3, pg. 14]. The sponsor may seek support in undertaking their review from NICE or other specialists in review/health economic methods. The evidence review and economic analysis provided by the sponsor is ‘validated’ by an EAG. NICE may also request the EAG undertake additional evidence collection in parallel to their critique of the sponsor submission. The MTEP manual notes that several approaches to synthesis may be considered appropriate dependent on the size and complexity of the evidence available. Synthesis approaches might include meta-analysis, network meta-analysis, sensitivity analysis and de novo modelling studies in the absence of data on clinical and system outcomes [3, pg. 18].

For complex diagnostic technology assessments routed through the DAP, an EAG undertakes a systematic review of the literature and considers data provided by sponsors and specialist (convened for the topic) Committee members. The EAG may also engage their own expert advisors [2, pg. 42]. Guidance is provided on the approach to searching which should begin with *“studies that follow patients from testing, through treatment to final studies (end to end studies)”* and *“if these studies exist, a systematic review of this evidence may remove the need for more extensive searches to identify evidence for model parameters”* [2, pg. 70].

If no end to end studies exist, a search for a range of evidence types is undertaken (systematic reviews, primary studies, expert opinion) covering a range of outcomes (test accuracy, test side effects, existing models of management and treatment following diagnosis, treatment effectiveness, impact of misdiagnosis, impact of test usage variation) to inform a ‘linked evidence’ approach [2, pg. 71]. We could not find specific methods for how reviewers should link evidence.

The existence of a good quality systematic review may mean a de novo systematic review is not necessary [2, pg. 80]. Guidance is provided for searching for test accuracy evidence [2, pg. 72-79] as well as for other outcomes [2, pg. 80].

Quality assessment approaches are detailed, particularly for test accuracy (use of STARD and QUADAS), but also for some other included study types. A list of types of bias applicable to test accuracy evidence is presented [2, pg. 74]. Extensive advice on synthesis of test accuracy studies is provided [2, pg. 72-79] and synthesis of other types of study design is also considered [2, pg. 80]. Where direct evidence on health outcomes is not available, guidance is provided for how to perform evidence linkage to model care pathways [2, pg. 85-89]. There is often a strong assumption that the effect of the test on patient outcomes occurs as a result of how the *“test is used in the diagnostic process*” and *“the impact of changed diagnostic information on subsequent disease management”* [2, pg. 86].

4. Recommended methods for evaluating the cost–effectiveness of diagnostic interventions: we looked for guidance on:

- *whether the HTA should involve any assessment of cost–effectiveness, and if so which methods are recommended (e.g. cost–utility, ‘cost–effectiveness’, cost–minimisation, budget impact analysis);*
- *how evidence should be incorporated into health economic models;*
- *how the clinical effectiveness review and cost–effectiveness study should link together (e.g. the extent to which clinical effectiveness review results are used to inform economic models);*
- *methods for carrying out linked evidence modelling, where this is different to health economic modelling*

### Methods for conducting health economics assessments

Health economics forms an integral part of the HTAs conducted by NICE. For less complex diagnostic technologies whose introduction into the NHS is likely to be cost neutral or cost saving (undergoing assessment by the MTEP), a cost-consequence analysis is considered appropriate and detail on cost-consequence models is provided [3, pg. 19-20].

For complex diagnostic technology assessments undertaken as part of the DAP, in the absence of existing high quality and relevant models of cost-effectiveness, a de novo model should be constructed and populated from the results of systematic reviews for each data input required. Modelling cost-effectiveness which is expanded on in some detail [2, 15]. Cost-effectiveness (specifically cost-utility) is the preferred type of economic evaluation type. The value of changes in HRQL (utilities) should be based on public preference using a choice-based method: EQ5D is the preferred measure. Where HRQL and mortality are not available for the specific condition under assessment, clinical outcomes measured in included studies should be mapped to QALYs. The conduct of the cost-effectiveness modelling is guided by a NICE reference case, [2, pg. 91]. Information on accuracy and treatment effectiveness should be obtained from systematic reviews. Linkages between diagnosis, (accuracy, direct and indirect test effects, the impact of the test on the diagnostic process), treatment (disease management) and final outcomes (effectiveness) must be specified and *“relevant data about those linkages needs to be obtained and reviewed”* [2, pg. 85].

Expert elicitation is suggested as a possible source of data, particularly for model parameters on which no evidence is found [2, pg. 71 and pg. 80]. Several special considerations for modelling are mentioned such as prognostic information, timing of tests, test sequence, test correlation, imperfect reference standard, and test thresholds [2, pg. 87-9]. There is no specific detail about either the basic modelling processes (methods for models should follow *“accepted guidelines”*) nor modifications to it to take into account these special considerations [2, pg. 97-98]. The methods guide specifies that *“simplified analyses”* may be possible under certain circumstances, for instance if *“high –quality end-to-end outcome studies are available”* but what is meant by a simple model structure is not detailed [2, pg. 84]. Uncertainty should be explored using sensitivity analyses: *“Probabilistic sensitivity analysis is the preferred method of investigating uncertainties”*, computable from the meta-analysis outputs or approximated [2, pg. 99].

### Special features noted

The DAP Guidance is structured like a handbook of methods as well as a medium for making process transparent.

NICE commit to undertaking updates of guidance 3 years after publication [5, pg. 3].

Special features of NICE guidance:

- Strong attention to process
- Prominent involvement of patients
- Extensive scoping phase involving stakeholders and care pathway research
- Emphasis on the use of modelling to assess whether the test is clinically and cost-effective – this is the means by which “evidence linkage” is achieved by NICE
- Final decision on recommendation relies heavily on the estimated cost-effectiveness (consistent with other guidance streams in NICE)
- Use of expert elicitation

### Questions to NICE

1. **Is the content of our summary above a fair and accurate reflection of your organisation’s processes and methods for undertaking HTAs of diagnostic tests?**
2. **Are there any publicly available documents we have missed that outline your processes for undertaking HTAs of tests?** We would like to provide a summary of organisations’ procedures for: topic referrals, pre–HTA scoping/topic development phase, involvement of stakeholders, post–review development of HTA into guidance**.**
3. **Are there any publicly available documents we have missed that outline the methods that should be used in an HTA of diagnostic tests?**
4. We are particularly interested in how test accuracy information is translated into conclusions about impact on patients’ outcomes. We note that your methods discuss linking evidence as part of “Evidence for assessment and evaluation”. **Is there more specific guidance on how a ‘linked evidence approach’ is achieved?**
5. We are aware that other organisations currently use GRADE for weighting the strength of different types of evidence. **Does GRADE have any part in your diagnostic technology appraisal process?**
6. We are also particularly interested in how HTA organisations identify the claims of diagnostic tests, which we note are sometimes referred to as ‘value propositions’ or ‘proposed benefits and harms’. **We can see that this is part of your scoping process, how do you incorporate that prioritisation of outcomes into your evaluation process?**

## Swedish Agency for Health Technology Assessment and Assessment of Social Services (SBU, Sweden)

### Sources used for information

[1] SBU Method Book (google translation into English), October 2020 [downloaded 10^th^ February 2021].

[2] Framework for Systematic Identification of Ethical aspects of Healthcare Technologies: The SBU Approach

[3] Organisation website (English version): <https://www.sbu.se/en> [last accessed 10th March 2021]

### HTA setting

Health technology assessment (HTA) in Sweden is led by its national body, the SBU. SBU together with SBU host a national HTA network where total 25 organisations participates (including the 21 regional HTA centres ) [https://www.sbu.se/en/collaboration/the-hta-network-sweden/].

1. Pre–evidence review processes for HTAs of tests: we looked for information on:

- *Where suggestions for which tests to review come from (referral source)*
- *Whether there are any entry criteria (e.g. regulatory conformity such as CE mark)*
- *Whether a formal scoping phase is recommended or mandated, and if so how the scope should be developed from the referred topic, and what the scope’s focus should be.*

### Processes for identifying diagnostic topics for HTA reports

### Proposals for SBU project topics come from various sources. The main sources today is The Ministry of Health and Social Affairs, but other sources are The SBU Scientific Advisory Committee, other Swedish government authorities, organisations and individuals [https://www.sbu.se/en/about-sbu/our-mandate/selection-of-topics/]. SBU undertakes preliminary investigations of research activity in topic field to establish the feasibility of drawing evidence-based, scientific conclusions, or instead identifying knowledge gaps that need to be addressed first [https://www.sbu.se/en/about-sbu/our-mandate/selection-of-topics/].The process for prioritisation of projects regarding diagnostic tests do not differ in any way from that of other technologies.

### Process for scoping HTA diagnostic topics:

SBU place emphasis on developing a well–defined PICO, which is modified to ‘PIRO’ for diagnostic toics (where I = index test, R=reference standard, Fact Box 3.1, p.6) and guidance is included on how to select a reference standard when there is no single capable test (p.14). Multiplicity of tests is recognised as important, as is the place of test in clinical pathway, where the role of the test using the categories suggested by Bossuyt and Lijmer is emphasised. Guidance on test–related outcomes is provided for measures of test accuracy (sensitivity, specificity, ROC, AUC) (p.14–18). The approach for defining the population is stated to be generic to all interventions, though SBU also direct reviewers to consider the role and position of the index test by comparison to any existing diagnostic strategy, highlighting that different roles of the index test produce differences in participant characteristics (p.13).

2. Review and post–review processes: we looked for information on:

- *What approach is used to develop evidence into guidance? e.g. by use of a committee to consider evidence reviews alongside any consultation processes*
- *How guidance is disseminated*
- *How patients or public, test developers, and/or experts are involved in the review process.*

### Process for involving stakeholders

The Method Book does not discuss how stakeholders are involved in the HTA process. The organisation’s website implies patients and healthcare users are key stakeholders at several points in the HTA process, particularly during scoping to formulate research questions, and at the end to analyse the implications of results from the HTA and assist in dissemination of knowledge to those affected by the topic [https://www.sbu.se/en/collaboration/patient-involvement/]. SBU collaborates actively during the entire HTA process with a large network of Swedish clinical experts, and these experts contribute to the review of clinical evidence and agreeing on findings and conclusions [https://www.sbu.se/en/impact/]. We did not find mention of test developers.

### Processes for developing evidence into guidance and disseminating guidance

Prior to approval and publication, assessments are reviewed by independent experts, SBU Quality assessment group, SBU Scientific Advisory Committees and the Board of Directors [https://www.sbu.se/en/about-sbu/our-mandate/our-products/]. The SBU Board of Directors and Advisory Committee approve the conclusions of every report [https://www.sbu.se/en/about-sbu/our-mandate/stages-in-assessment-work/]. We also note that guidance is disseminated readily with reviews available in Swedish and English on the SBU website, and a widespread dissemination strategy covering mass–media, conferences and educational programmes, interactive sessions and collaboration with relevant government agencies [https://www.sbu.se/en/impact/].

3. Recommended methods for performing a clinical effectiveness review of a diagnostic test: we looked for guidance on:

- *whether the HTA involves collection of evidence for review of clinical effectiveness;*
- *who collects the information and how this is funded;*
- *which evidence should be sought for tests, and using which search methods;*
- *methods to appraise the quality of evidence, and how to incorporate these into conclusions;*
- *summarising and synthesizing evidence*
- *methods to judge the relative strength and importance of the evidence? (e.g. GRADE)*

### Overview of guidance on methods for Health Technology Assessments of tests

The SBU has produced a method guide/Handbook for undertaking HTA, which is used by the regional HTA centres as well as other Agencies in Sweden. It was updated in October 2020 and is available as a html version in Swedish language only on the SBU website . [1]. Methods for reviewing diagnostic tests are spread throughout the Method Book, as dedicated subsections of key HTA methods chapters:

Ch 3.3 Format of questions about diagnostic tests

Ch 6.2 Risk of bias in studies on diagnostic reliability

Ch 8.2 Meta–analysis for diagnostic reliability

Ch 9.10 (GRADE) Diagnostic reliability

At SBU, an assessment can either be an HTA report i.e includes both a systematic review and the certainty of evidence as well a health economics analysis and an assessment of Ethics, where relevant or can only consist of a systematic review. SBU have developed a framework for systematically identifying the ethical aspects of any health technology [2], without specific tailoring for diagnostic test interventions.

### Methods for reviewing evidence of diagnostic tests:

Scientific evidence is identified using general methods for systematic reviews (PRISMA statement chapter 2), with no specific advice for diagnostic questions other than to use the ‘PIRO’ to create the search strategy (p.29). SBU’s assessments are independent [https://www.sbu.se/en/about-sbu/our-mandate/], performed by professional practitioners, academics, patients/user representatives and SBU staff [https://www.sbu.se/en/about-sbu/our-mandate/our-products/]. Details outlining how HTAs are funded were not found.

SBU focus on collecting direct evidence for the performance and impact of tests. Direct evidence of health outcome data from RCTs is considered ideal, however SBU acknowledge that this type of evidence is very rare and so focus their methods guidance on reviewing diagnostic test accuracy, which they term ‘diagnostic reliability’ (p.12). Once evidence has been selected for inclusion, SBU recommends its methodological quality and applicability are assessed using the QUADAS–2 instrument with appropriate tailoring. There is no guidance for how to incorporate quality assessments into the review’s conclusions. The approach to data synthesis should be meta–analysis (where possible), for which general methods are provided (chapter 8.2).

SBU has a clear philosophy that a test’s accuracy is necessary but insufficient to determine the effectiveness of a new test (p.12). This is reflected in their guidance on interpreting diagnostic meta–analysis results, which they recommend should include a discussion of the consequences of false negative and false positive results (sec 8.2.3). Their methodology does not appear to apply a ‘linked evidence’ approach to model health outcomes using test accuracy data.

We did not find guidance for which HTA methods to use should a review identify evidence for a test’s impact on to health outcomes, for example test–treatment RCTs or observational clinical impact studies. Guidance is provided for appraising and analysing RCTs and non–randomised studies in general (chapter 6.1), for example directing the reviewer to use Cochrane Collaboration RoB 2 tool for RCTs and ROBINS–I for non–randomised studies (p.45) although no tailoring to trials of diagnostic test interventions was found.

The relative strength and importance of evidence is assessed using the GRADE framework for tests (sec 9.10), an approach modified by SBU, although the type of modification is not reported. While GRADE downgrades the strength of accuracy evidence for informing the likely health impacts of a test, evidence included in HTAs of tests whose primary outcome is test accuracy are not penalised (sec 9.10).

4. Recommended methods for evaluating the cost–effectiveness of diagnostic interventions: we looked for guidance on:

- *whether the HTA should involve any assessment of cost–effectiveness, and if so which methods are recommended (e.g. cost–utility, ‘cost–effectiveness’, cost–minimisation, budget impact analysis);*
- *how evidence should be incorporated into health economic models;*
- *how the clinical effectiveness review and cost–effectiveness study should link together (e.g. the extent to which clinical effectiveness review results are used to inform economic models);*
- *methods for carrying out linked evidence modelling, where this is different to health economic modelling*

### Methods for conducting health economics assessments

Health economics forms an integral part of HTAs at the SBU, and their Handbook outlines their general approach consisting of systematic review of economic evidence (p.116–7), as well as de–novo economic modelling (p.117–124) and budget impact analysis (p.125). A variety of approaches can be selected (including cost-minimisation, cost–consequence, cost-effectiveness, cost-utility and cost-benefit), with the choice of method determined by the question and availability of data (p.119). We did not find information on whether adaptations are needed for diagnostic tests.

### Special features noted

- Preference for the term ‘diagnostic reliability’, rather than ‘diagnostic accuracy’
- Use of GRADE approach, modified by SBU, to weight different types of evidence on tests
- Systematic consideration of ethical considerations for all health technologies, including tests

### Questions to SBU

1. **Is the content of our summary above a fair and accurate reflection of your organisation’s processes and methods for undertaking HTAs of diagnostic tests?** We have done some modifications (incorporated as requested)
2. **Are there any publicly available documents we have missed that outline your *processes* for undertaking HTAs of tests?** We would like to provide a summary of organisations’ procedures for: topic referrals, pre–HTA scoping/topic development phase, involvement of stakeholders, post–review development of HTA into guidance**.** Note that is available in English. As you have seen we have updated our handbook, which was published in October 2020. Unfortunately, it is available only in Swedish. However, SBU produces systematic reviews according to the PRISMA statement
3. **Are test developers, patients and experts involved in any part of the HTA process?** We have noted that user/patient representatives and clinicians are involved in various stages of producing SBU assessments. Are test developers involved at any stage? For example during the topic scoping/question development phase, collection of evidence, interpretation of evidence, or production of final guidance. Our project teams always consist of staff from SBU and 2-6 experts in the field. They take part in all steps. Patients can be involved in some parts of the project, but it varies depending on several factors. Test developers are not included
4. **Are there any publicly available documents we have missed that outline the *methods* that should be used in an HTA of diagnostic tests?** Do not think so , but difficult to say when the reference list was missing
5. We are particularly interested in how test accuracy information is translated into conclusions about impact on patients’ outcomes. We note that some people refer to this as evidence linkage or ‘linked–evidence’ modelling. **Your methods imply to us that you accept only direct evidence of clinical effectiveness (measured in a clinical study), and do not perform modelling to estimate the health impacts of diagnostic tests when your evidence–base is restricted to diagnostic reliability studies. Is this correct?** Yes
6. **We note GRADE is an important part of your process for weighting the strength of different types of evidence, however there seems to be a problem with our translation:**

Chapter 9.10: “GRADE focuses on outcomes that are important to the patient or client, ie the value of a method improving health or reducing problems. GRADE therefore believes that sensitivity and specificity are surrogate measures for the important outcome. The results thus have reduced portability. *In the event that the SBU research question applies to the diagnostic reliability of the method is, it wants to say when our own primary outcome measure, the sensitivity and specificity, however, made no deduction for lack of transferability*.” **Would you be able to provide us with a better translation for the sentence in italics so we can better understand your methods?** Suggested alternative **“**GRADE focuses on outcomes that are important to the patient or client, ie the value of a method improving health or reducing problems. GRADE therefore believes that sensitivity and specificity are surrogate measures for the important outcome. This affects the directness. However, in projects where SBU evaluate diagnostic accuracy , i.e the main outcome is sensitivity and specificity, this is not a problem/factor.”

1. We are also particularly interested in how HTA organisations identify the claims of diagnostic tests, which we note are sometimes referred to as ‘value propositions’ or ‘proposed benefits and harms’. **Do you have a particular process or approach for determining the claims of a diagnostic test selected for HTA?** No

## National Health Care Institute (Zorginstituut Nederland) (ZIN, Netherlands)

### Sources used for information

[1] ***Medical tests (assessment of established medical science and medical practice)***. Approved by CVZ on 20th January 2011. Publication number 293. [downloaded 23^rd^ March 2021]

[2] **Assessment of ‘established medical science and medical practice’.** Zorginstituut Nederland. Beoordeling stand van de wetenschap en praktijk. Diemen, 2015. [downloaded 22^nd^ March 2021].

[3] ***Assessment of ‘established medical science and medical practice’: a technical modification.*** Final version, 23^rd^ April 2019. Series number 2019003598. [downloaded 23^rd^ March 2021].

[4] ***Guideline for economic evaluations in healthcare.*** Final version, 16^th^ June 2016. Series number 2016077622. [downloaded 22^nd^ March 2021].

[5] ***Tasks of the National Health Care Institute.*** Zorginstituut Nederland’s web page, available from: <https://english.zorginstituutnederland.nl/about-us/tasks-of-the-national-health-care-institute> [last accessed 6^th^ May 2021].

[6] ***Working method for the Zinnige Zorg*** (Appropriate Care Programme), National Health Care Institute, August 2018. [downloaded 23^rd^ March 2021].

[6] ***Appropriate Care***. Zorginstituut Nederland’s web page, available from: <https://english.zorginstituutnederland.nl/zinnige-zorg> [last accessed 6^th^ May 2021].

### HTA setting

Health technology assessment (HTA) in the Netherlands is carried out by the Dutch National Health Care Institute (Zorginstituut) which is “*an advisory and implementing organisation for two statutory health insurance schemes: the Health Insurance Act (Zorgverzekeringswet, Zvw) and the Long­Term Care Act (Wet Langdurige Zorg, Wlz)*”[5]. The Institute “*assesses whether diagnostics and (therapeutic) interventions are being deployed in a patient-oriented, effective and cost-effective manner*.” Its guiding principle is that “*Every citizen must be able to count on receiving good health care. No more and no less than is necessary, while also avoiding unnecessary costs*.” [6, p.2]. General basic health insurance only includes care (including medical tests) that is considered effective according to the ‘established medical science and medical practice’ [1].

1. Pre–evidence review processes for HTAs of tests: we looked for information on:

- *Where suggestions for which tests to review come from (referral source)*
- *Whether there are any entry criteria (e.g. regulatory conformity such as CE mark)*
- *Whether a formal scoping phase is recommended or mandated, and if so how the scope should be developed from the referred topic, and what the scope’s focus should be.*

### Processes for identifying diagnostic topics for HTA reports

In order to promote good care, The Dutch National Health Care Institute carries out systematic assessment of specific topics following a stepwise approach termed ‘circle of improvement’ which includes: Screening phase, in-Depth Analysis phase, Implementation phase and Evaluation phase. In the Screening phase a number of topics that have the potential for improving the quality and effectiveness of care are selected for in-depth analysis. The topics could be identified by the Institute or requested: “*CVZ*^[[1]](#footnote-1)^ *does regularly assess – upon request or at CVZ’s own initiative – whether (innovative) care should (actually) be included in the basic package. This could be for a variety of reasons*.” [1, p.8]. The topics are then presented to the parties in healthcare and to the Minister of Health Welfare and Sport.

The proposals for evaluation of ‘test-plus-treatment’ strategies follow the same process. However, *Section 5.h.* of *Medical Tests (assessment of established medical science and medical practice)* states that, in relation to professionals and manufacturers, “*whoever asks for CVZ’s opinion over a test-plus-treatment-strategy must substantiate their reques*t”. Applicants are expected to describe the claim for the proposed ‘test-plus-treatment strategy’ in terms of health outcomes and to provide relevant data including data on the analytical and diagnostic accuracy of the test [1, p.17, 29-30].

### Process for scoping HTA diagnostic topics

The point of departure in the assessment of medical tests is that the test needs to lead to health benefits for patients (termed clinical utility of the test). Therefore, the focus of assessment is the proposed test-plus-treatment strategy, which is compared to current (best) practice. Here, the term ‘treatment’ is defined broadly to include “*all interventions that will be used based on the test and that will have effect on the final outcome for the patient*” [1, p.15]. In the scoping exercise, the PICO parameters of the assessment are defined:

- P: the patients and the setting in which they are being tested
- I: the test-plus-treatment strategy being investigated
- C: the comparative test-plus-treatment strategy (the best/usual strategy)
- O: the appropriate outcome indicators related to the patient’s health.

The above should be based on the claim(s) made for the test. The patients and the setting within which the new test is to be used should be precisely defined. The role of the test in the test-plus-treatment strategy (as a replacement, add-on or triage test) should be clearly formulated. Appropriate outcome indicators should be selected avoiding intermediate outcomes (e.g. the therapy choice of doctors) which can lead to erroneous conclusion about the utility of the test-plus-treatment strategy [1].

2. Review and post–review processes: we looked for information on:

- *What approach is used to develop evidence into guidance? e.g. by use of a committee to consider evidence reviews alongside any consultation processes*
- *How guidance is disseminated*
- *How patients or public, test developers, and/or experts are involved in the review process.*

### Process for involving stakeholders

The Dutch National Health Care Institute places particular importance on transparency and stakeholder involvement. The *Working method for the Zinnige Zorg (appropriate care) Programme* (2018) states that “*Parties in health care are involved throughout the entire process*” [6, Section 1.4]. The documents specifically mention the involvement of professionals (through their scientific organisations), patients (through their associations) and health insurers. They participate by attending consultations via umbrella arrangements; by being given the opportunity to supervise the research of external research bureaus; and, by commenting on the draft versions of the reports.

Professionals and patients are also involved in the development of the PICO questions, so they can share their practical knowledge and experience [2, p.2]. For each assessment advice is obtained from a committee of independent external experts, and the Scientific Advisory Committee (Wetenschappelijk Adviesraad) [2, p.3]. External experts are usually invited to comment on the draft report, but in some cases they could also be involved “*during the design phase in order to obtain an (initial) critical assessment*” [1, p.30]. Test developers/manufacturers are specifically mentioned only in relation to substantiating their request for assessment of a test-plus-treatment strategy, by contributing specific information (see above).

### Processes for developing evidence into guidance and disseminating guidance

As mentioned above, the process of assessment includes the following four phases: Screening phase, in-Depth Analysis phase, Implementation phase and Evaluation phase. The in-Depth Analysis results in the so-called Improvement Report, which “*states which improvements in care and in health the Zorginstituut feels are possible, in respect of both content and amount, and provides an estimate of the total sum of costs involved (budget impact)*” [6, p.6]. The Improvement report is sent to the parties in health care and to the Minister of Health Welfare and Sport. The implementation of the recommended improvements is “primarily a task for the parties in health care: patients, care professionals, institutions and health insurers” based on the agreements made in the in-Depth phase. The role of the Zorginstituut is to advise and provide support; to place “*action points from the Improvement Report that relate to quality standards and measuring instruments on the Multi-Year Agenda*” and to monitor the progress (Evaluation phase) [6, p.6]. The above is the general process followed by the Zorginstituut and is not specific to test evaluations.

3. Recommended methods for performing a clinical effectiveness review of a diagnostic test: we looked for guidance on:

- *whether the HTA involves collection of evidence for review of clinical effectiveness;*
- *who collects the information and how this is funded;*
- *which evidence should be sought for tests, and using which search methods;*
- *methods to appraise the quality of evidence, and how to incorporate these into conclusions;*
- *summarising and synthesizing evidence*
- *methods to judge the relative strength and importance of the evidence? (e.g. GRADE)*

### Overview of guidance on methods for Health Technology Assessments of tests

The Dutch National Health Care Institute’s method for HTA of medical tests is presented in the *Medical tests (assessment of established medical science and medical practice)* report [1]. Further guidance confirming the main principles of test evaluation (e.g. the focus on clinical utility and the comparison of ‘new’ and ‘old’ test-plus-treatment strategies) is provided in the 2015 update *Assessment of ‘established medical science and medical practice’* [2]. The latter also states that “*We are gradually starting to use the GRADE method in assessing tests too.*” [2, p.4]. A separate guideline, *Assessment of ‘established medical science and medical practice’: a technical modification* (2019), deals with the special case of a technical modification of an intervention that is already covered by the general basic health insurance and may or may not need additional assessment [3].

### Methods for reviewing clinical evidence of diagnostic tests

Medical tests are considered an integral part of healthcare interventions, and as such should meet the criteria for ‘*established medical science and medical practice*’ if they are to be covered by the general basic health insurance, as stipulated in the Dutch Health Insurance Act. The point of departure in the Dutch National Health Care Institute’s method for HTA of medical tests is that “*a medical test can only be regarded as complying with ‘established medical science and medical practice’ if there is evidence, or it has been made plausible, that using the test leads to health benefits for patients*” [1, p.14-15). This is termed ‘clinical utility of the test’ and its assessment involves the assessment of the whole test-plus-treatment strategy (where ‘treatment’ is defined broadly as all the interventions that follow). It is also acknowledged that “*the health outcomes related to patients are not the only effects of tests*” [1, p.18] and other aspects, such as deployment means (techniques, personnel, money) and undesired effects should also be considered in the evaluation.

The process of HTA begins with 1) formulating a research question in terms of PICO (patient, intervention, comparator and outcome); 2) then the availability of direct evidence, preferably in the form of RCTs, is determined; 3) if no direct evidence is found, or it is insufficient/inadequate for the specific claim(s), the next step is to determine “*whether indirect evidence can sufficiently demonstrate the clinical utility of the proposed test-plus-treatment-strategy*”; 4) the final stage of the process is the formulation of conclusions on the basis of which the Dutch Health Care Insurance Board can make a decision about whether or not to include a given test (-plus-treatment strategy) in the health insurance package [1, p. 23-24].

Direct evidence refers to studies that compare directly, in the same group of patients the proposed test-plus-treatment strategy and the current (best) practice, and report appropriate health outcomes that reflect the claim(s) made for the new strategy. RCTs are preferred, direct evidence from studies with a lower level of evidence can also be acceptable. However, it is acknowledged that direct evidence is rarely available and is not always necessary [1, p. 24].

In this case a ‘*comparative analysis framework for indirect evidence’* should be constructed [1, p. 25-29]. The purpose of the framework is to “*help to reply to the question of whether the clinical utility of a test-plus-treatment-strategy has been demonstrated or made plausible*.” [1, p. 23]. The process includes the following steps: 1) determine the role of the test (as a replacement, add-on or triage test); 2) determine the target population/stage in the care process; 3) determine claims in terms of health outcomes; 4) define the comparator (e.g. current [best] test-plus-treatment strategy); 5) set out the new test-plus-treatment strategy; 6) identify critical comparisons (advantages and disadvantages) of the new strategy; 7) identify and prioritise all differences between tests, in order to be clear about the questions posed; 8) carry out literature research to collect replies to the questions; 9) determine whether the questions have been answered sufficiently or crucial data are lacking; 10) formulate a conclusion [1, p. 28-29].

The document mentions specific aspects of conducting a systematic review of the evidence necessary to answer the questions generated within the comparative framework (e.g. systematic searches, methodological quality assessment using QUADAS-2). However, no technical guidance specific to test evaluations or, more generally, to health interventions, is provided in the reviewed documents.

4. Recommended methods for evaluating the cost–effectiveness of diagnostic interventions: we looked for guidance on:

- *whether the HTA should involve any assessment of cost–effectiveness, and if so which methods are recommended (e.g. cost–utility, ‘cost–effectiveness’, cost–minimisation, budget impact analysis);*
- *how evidence should be incorporated into health economic models;*
- *how the clinical effectiveness review and cost–effectiveness study should link together (e.g. the extent to which clinical effectiveness review results are used to inform economic models);*
- *methods for carrying out linked evidence modelling, where this is different to health economic modelling*

Health economics forms an integral part of the HTAs conducted by the Dutch National Health Care Institute and their general approach is detailed in the *Guideline for economic evaluations in healthcare* [4]. Although different analytic techniques, such as CUA, CEA and CMA, are briefly discussed in the first section of Chapter 2, the main focus is on CUA, with additional chapter on Budget Impact Analysis (Chapter 5). Chapter 6 provides additional information on applications other than pharmaceutical care, including Diagnostics [4, Section 6.2, p.38]. The recommended approach for an economic evaluation of a diagnostic method is to follow the reference case analysis as strictly as possible paying attention to the following specific aspects: sensitivity and specificity of the test; synthesis and quality of test accuracy data, using QUADAS-2 as a method of assessment; target population and the associated incidence and prevalence of the target condition; the consequences of a wrong diagnosis, considering both false negative and false positive results; and the role of the test in the test-and-treatment strategy. It is acknowledged that “*Diagnostic methods can be appreciated in more ways than only on the basis of the medical outcomes as established with the EQ-5D*.” [4, p.38] and that other value components could be identified by directly consulting patients. For further information, the reader is referred to the manual of the NICE Diagnostics Assessment Programme.

### Special features noted

- A framework for comparative analysis when no direct evidence is available.
- A framework for assessment of a technical modification of an intervention whose effectiveness has been established or is not open to discussion [3].

### Questions to ZIN

1. Is the content of our summary above a fair and accurate reflection of your organisation’s processes and methods for undertaking HTAs of diagnostic tests?
2. Are there any publicly available documents we have missed that outline your processes for undertaking HTAs of tests *in more detail*? Additional documents detailing the procedures for topic referrals, pre–HTA scoping/topic development phase, involvement of stakeholders, post–review development of HTA into guidance.
3. Are there any additional documents on the involvement of test developers, patients and experts in any part of the HTA process?
4. Are there any publicly available documents we have missed that outline the methods that should be used in an HTA of diagnostic tests?
5. We are particularly interested in how test accuracy information is translated into conclusions about impact on patients’ outcomes. We note that in the absence of direct evidence for the effectiveness of a test–plus–treatment strategy (such as RCT evidence), you evaluate indirect evidence by means of a ‘comparative analysis framework’. **Do such methods include modelling to estimate the test–treatment effect? And, are there any publicly available documents, not included in the summary, that describe in more detail the process and methods for linking indirect evidence to produce the final conclusions (for instance, more detailed description of the use of ‘linked–evidence’ modelling)?**
6. We are also particularly interested in how HTA organisations identify the claims of diagnostic tests, which we note are sometimes referred to as ‘value propositions’ or ‘proposed benefits and harms’. We note that the general process of identifying the claims of diagnostic tests is described in the identified documents. **Are there any additional documents that describe this process and the related procedures in more detail?**

1. the Dutch Health Care Insurance Board [↑](#footnote-ref-1)
