# Supplementary material for: Health technology assessment of diagnostic tests: a state of the art review of methods guidance from international organizations
Source: Int J Technol Assess Health Care. 2023 Feb 21;39(1):e14. doi: 10.1017/S0266462323000065 (PMC7614237; doi:10.1017/S0266462323000065)
Supplement: Supplementary file 1 [file S0266462323000065sup001.zip › S0266462323000065sup003.docx]

**Supplementary Table 1: Eligible HTA organisations with methods documents (N=41)**

| **Country** | **HTA organisation** | **Acronym** | **Methods documents?** |
| --- | --- | --- | --- |
| Argentina | Comisión Nacional de Evaluación de Tecnologías de Salud [National Commission for Health Technology Assessment] | CONETEC | Yes |
| Argentina | Instituto de Efectividad Clinica Y Sanitaria [Institute for Clinical Effectiveness and Health Policy] | IECS | Yes |
| Australia | Medical Services Advisory Committee | MSAC | Key organisation* |
| Austria | Austrian Institute for Health Technology Assessment | [AIHTA](https://eunethta.eu/aihta) | Yes** |
| Austria | Donau-Universität Krems [Danube University Krems] | DUK | Yes** |
| Austria | Gesunheit Österreich GmbH [Health Austria] | GOG/BIQG | Yes** |
| Austria | University for Health Sciences, Medical Informatics and Technology (UMIT), Institute of Public Health, Medical Decision Making and Health Technology Assessment | UMIT | Yes** |
| Belgium | Federaal Kenniscentrum/Centre fédéral d'expertise [Belgian Health Care Knowledge Centre] | KCE | Yes |
| Brazil | Comissão Nacional de Incorporação de Tecnologias no Sistema Único de Saúde [National Commission for the Incorporation of Technologies in the Unified Health System] | CONITEC | Yes |
| Canada | Canadian Agency for Drugs and Technologies in Health | CADTH | Key organisation* |
| Canada | Health Quality Ontario | HQO | Yes |
| Canada | Institut National d’Excellence en Santé et en Services Sociaux [National Institute of Excellence in Health and Social Services] | INESSS | Yes |
| China (Taiwan) | Center for Drug Evaluation | CDE | Yes |
| Colombia | Instituto de Evaluación Tecnológica en Salud [Institute of HealthTechnology Evaluation] | IETS | Yes |
| Denmark | Social sundhed & arbejdsmarked [Social & Health Services and Labour Market] | DEFACTUM | Yes |
| El Salvador | Ministerio de Salud [Ministry of Health El Salvador] | MoH El Salvador | Yes |
| Estonia | Tervisetehnoloogia Hindamise,  Tartu Ülikool [Health Technology Assessment, University of Tartu] | [TTH](https://eunethta.eu/uta) | Yes |
| France | Haute Autorité de Santé [National Authority for Health] | HAS | Yes |
| Germany | docuInstitut für Qualität und Wirtschaftlichkeit im Gesundheitswesen [The Institute for Quality and Efficiency in Healthcare] | IQWiG | Key organisation* |
| India | Health Technology Assessment in India | HTAIn | Yes |
| Indonesia | Indonesia Health Technology Assessment Committee | InaHTAC | Yes |
| Ireland | Health Information and Quality Authority | HIQA | Yes |
| Italy | Agenzia Nazionale per i Servizi Sanitari Regionali [National Agency for Regional Health Services] | AGENAS | Yes |
| Italy | Agenzia Sanitaria e Sociale Regionale [Regional Agency for Health and Social Care] | ASSR | Yes |
| Japan | Center for Outcomes Research and Economic Evaluation for Health | C2H | Yes |
| Malaysia | Malaysian Health Technology Assessment Section | MaHTAS | Yes |
| Netherlands | Zorginstituut Nederland [National Health Care Institute] | ZIN | Key organisation* |
| Philippines | Sentro ng Pagsusuri ng Teknolohiyang Pangkalusugan (STEP) | STEP | Yes |
| Poland | Agencja Oceny Technologii Medycznych I Taryfikacji [Agency for Health Technology Assessment and Tariff System] | AOTMiT | Yes |
| Portugal | Sistema Nacional De Avaliação De Tecnologias De Saúde (Autoridade Nacional do Medicamento e Produtos de Saúde) [National Authority of Medicines and Health Products] | [SiNATS (INFARMED)](https://eunethta.eu/infarmed) | Yes |
| Singapore | Agency for Care Effectiveness | ACE | Yes |
| South Korea | National Evidence-based healthcare Collaborating Agency | NECA | Yes |
| Spain | Avaliación de Tecnoloxías Sanitarias de Galician [Galician Agency for Health Technology Assessment] | AVALIA–T | Yes |
| Spain | Servicio de Evaluación de Tecnologías Sanitarias [Basque Office for Health Technology Assessment] | OSTEBA | Yes |
| Sweden | Statens Beredning För Medicinsk Och Social Utvärdering [Swedish Agency For Health Technology Assessment And Assessment Of Social Services] | SBU | Key organisation* |
| Thailand | Health Intervention and Technology Assessment Program | HITAP | Yes |
| UK | National Institute for Health and Care Excellence | NICE | Key organisation* |
| USA | Agency for Healthcare Research and Quality | AHRQ | Key organisation* |
| EU | European Network for Health Technology Assessment | EUnetHTA | Yes |
| Global | World Health Organisation | WHO | Yes |
| Global | Grading of Recommendations Assessment, Development and Evaluation | GRADE | Yes |

The table is ordered by country and acronym. The three organisations not specific to a country are listed last.

*41 organisations contributed eligible methods documents. The seven key organisations were included in Stage 1 in-depth examination while the remaining 34 were included in Stage 2 rapid interrogation of methods guides from the remaining organisations using the key themes generated in Stage 1.

** One document was jointly produced by all four Austrian organisations [GOG, AIHTA, UMIT, DUK] creating 38 unique sources.
