# Supplementary material for: Health technology assessment of diagnostic tests: a state of the art review of methods guidance from international organizations
Source: Int J Technol Assess Health Care. 2023 Feb 21;39(1):e14. doi: 10.1017/S0266462323000065 (PMC7614237; doi:10.1017/S0266462323000065)
Supplement: Supplementary file 1 [file S0266462323000065sup001.zip › S0266462323000065sup004.docx]

**Supplementary Table 2: Remit, capacity and sources used for 7 HTA organisations included in Stage 1 assessment**

| **Stage 1 organisation (acronym, country)** | **HTA remit** | **Document types and sources** | **HTA setting** |
| --- | --- | --- | --- |
| **Agency for Healthcare Research and Quality (AHRQ, USA)** | All healthcare interventions, including all medical tests. | **Methods guide**^1^ specific to medical tests.  Other sources:  [1]. ***Agency for Healthcare Research and Quality: A Profile.*** Content last reviewed March 2018. Agency for Healthcare Research and Quality, Rockville, MD. https://www.ahrq.gov/cpi/about/profile/index.html [last accessed 10th June 2021]  [2]. ***EHC Program Topic Nomination and Selection***. EHC AHRQ web page, available from: https://effectivehealthcare.ahrq.gov/about/epc/nomination/ [last accessed 20th July 2020].  [3]. ***Evidence–based Practice Centers***. EHC AHRQ web page, available from: https://effectivehealthcare.ahrq.gov/about/epc [last accessed 24th July 2020].  [4]. ***About the Evidence Synthesis Process.*** EHC AHRQ web page, available from: https://effectivehealthcare.ahrq.gov/about/epc/evidence-synthesis [last accessed 24th July 2020].  [5]. ***Evidence-based Practice Center Editorial Review Process.*** EHC AHRQ web page, available from: https://effectivehealthcare.ahrq.gov/about/epc/review [last accessed 24th July 2020].  [6]. ***Evidence-based Practice Center Evidence Report Surveillance Process.*** EHC AHRQ web page, available from: https://effectivehealthcare.ahrq.gov/about/epc/update [last accessed 24th July 2020].  [7]. ***Methods Guide for Medical Test Reviews.*** AHRQ Publication No. 12-EC017. Rockville, MD: Agency for Healthcare Research and Quality; June 2012. Downloaded 20th July 2020.  [8]. ***EPC Methods Guide for Comparative Effectiveness Reviews***. Available at https://effectivehealthcare.ahrq.gov/products/cer-methods-guide [last accessed 6th June 2021]. | AHRQ is the lead Federal agency for improving the safety and quality of the US’s health care system, including HTA which falls within the remit of AHRQ’s Effective Health Care Program (EHC) [1]. AHRQ conduct ‘comparative effectiveness reviews’ [8], which are conceived as systematic reviews of the evidence on tests, possibly augmented by modelling. AHRQ provides technology assessments to inform the US Centers for Medicare and Medicaid Services (CMS) coverage decisions. |
| **Canadian Agency for Drugs and Technology in Health (CADTH, Canada)** | All healthcare interventions, including all medical tests. | **Methods guide**^2^ for economic evaluations for all interventions, with reference to tests throughout and an appendix for codependent tests^3^  *Clinical Effectiveness Review methods not publicly available.  Other sources:  [1] ***About the Health Technology Assessment Service***, CADTH website: https://www.cadth.ca/about-cadth/what-we-do/products-services/hta (Last Updated 15th June 2021) [last accessed 16th June 2021].  [2] ***Overview of HTA and OU Medical Devices and Clinical Interventions***. [downloaded 24th July 2020]. Ottawa: CADTH.  [3] ***Health Technology Assessment and Optimal Use: Medical Devices; Diagnostic Tests; Medical, Surgical, and Dental Procedures.*** CADTH Topic Identification and Prioritization Process. November 2015 (Version 1.0). Ottawa: CADTH. [downloaded 24th July 2020].  [4] ***Guidelines for the Economic Evaluation of Health Technologies: Canada. 4th ed.*** Ottawa: CADTH; 2017 Mar. [downloaded 24th July 2020].  [5] ***Guidelines for the Economic Evaluation of Health Technologies: Canada. 4th ed. Appendix — Specific guidance for treatments with companion diagnostics.*** Ottawa: CADTH; 2019 Sep. [downloaded 24th July 2020].  [6] ***Guidelines for Authors of CADTH Health Technology Assessment Reports.*** Revised May 2003. Ottawa: CADTH. [downloaded 23rd September 2020].  [7] ***Health Technology Expert Review Panel***, CADTH website: https://www.cadth.ca/collaboration-and-outreach/advisory-bodies/health-technology-expert-review-panel (Last Updated 16th June 2021) [last accessed 16th June 2021].  [8] ***Device Advisory Committee***, CADTH website: https://www.cadth.ca/device-advisory-committee-dac (Last Updated 16th June 2021) [last accessed 16th June 2021].  [9] ***CADTH Health Technology Expert Review Panel Terms of Reference.*** March 2020. Ottawa: CADTH. [downloaded 23rd September 2020].  [10] ***CADTH Health Technology Expert Review Panel Deliberative Framework***. May 2015. Ottawa: CADTH. | CADTH is an independent, not-for-profit organization responsible for providing health care decision-makers with objective evidence to help make informed decisions about the optimal use of health technologies. In addition to evidence, it also provides advice, recommendations, and tools. HTA products include comprehensive HTA reports, technology reviews (custom HTAs *“that contain some but not all of the elements of a traditional HTA (for example, an economic review without a clinical component”.* [1] |
| **Institute for Quality and Efficiency in Health Care (Institut für Qualität und Wirtschaftlichkeit im Gesundheitswesen (IQWiG, Germany)** | All healthcare interventions, including all medical tests. | **Methods guide**^4^ for all interventions, with test–specific chapters (2020)  Other sources:  [1] **General methods (Version 5.0, 10th July 2017).** English translation of *Allgemeine Methoden*. (Available from: https://www.iqwig.de/en/about-us/methods/methods-paper/). [pdf downloaded 24th July 2020].  [2] **General Methods (Version 6.0, 5th Nov 2020).** Informal translation of Final Draft of *Allgemeine Methoden*. (Available from: https://www.iqwig.de/en/about-us/methods/methods-paper/). [pdf downloaded 12th December 2020].  [3] **Basic principles.** (Available from: https://www.iqwig.de/en/methods/basic-principles.3314.html]. (Last accessed 24th July 2020).  [4] **Contracting agencies and funding of IQWiG.** [available from: https://www.iqwig.de/en/about-us/responsibilities-and-objectives-of-iqwig/contracting-agencies-and-funding.2951.html] (Last accessed 24th July 2020).  [5] **Institute structure.** (Available from: https://www.iqwig.de/en/about-us/institute-structure.2953.html). (Last accessed 24th July 2020).  [6] **About us – An introduction to IQWiG.** (Available from www.IQWiG.de). [pdf downloaded 24th July 2020].  [7] **Non–drug interventions: responsibilities of the department.** (Available from: https://www.iqwig.de/en/about-us/institute-structure/departments/non-drug-interventions.2976.html). (Last accessed 24th July 2020). | IQWiG is Germany’s national HTA body, responsible for providing HTA findings to the Federal Joint Committee (G–BA) to inform decisions on reimbursement. Its tasks encompass the production of HTAs [1]. |
| **Medical Services Advisory Committee (MSAC, Australia)** | All non–pharmaceutical interventions.  ‘Investigative services’ include all types of medical testing, defined as: “*a service that generates clinically-relevant information about the individual to whom the service is rendered*” [4, p.8]. | **Methods Guide**^5^ for health technologies other than medicines, with test–specific chapters and specific considerations for tests throughout.  Other sources:  [1] Medical Services Advisory Committee. 2021. ***Guidelines for preparing assessments for the Medical Services Advisory Committee.*** [Online]. Version 1.0. [Last accessed 16th June 2021]. Available from: http://www.msac.gov.au/internet/msac/publishing.nsf/Content/Documents-for-Applicants-and-Assessment-Groups  [2] Medical Services Advisory Committee (MSAC) ***Reform Implementation.*** ***2016. Process Framework***. [Online]. Version 1.0. [Last accessed 20th July 2021]. Available from: http://www.msac.gov.au/internet/msac/publishing.nsf/Content/msac-process-framework  [3] Australian Government Department of Health. 2021. ***Application Form.*** [Online]. Version 2.4 [Last accessed 7th July 2021]. Available from: http://www.msac.gov.au/internet/msac/publishing.nsf/Content/Documents-for-Applicants-and-Assessment-Groups  [4] Australian Government Department of Health. 2021***. MSAC Application Form Instructions.*** [Online]. Version 1 [Last accessed 7th July 2021]. Available from: http://www.msac.gov.au/internet/msac/publishing.nsf/Content/Documents-for-Applicants-and-Assessment-Groups  [5] Australian Government Department of Health. 2021. ***PICO Confirmation (To guide a new application to MSAC***). [Online]. Version 2.0 [Last accessed 7th July 2021]. Available from: http://www.msac.gov.au/internet/msac/publishing.nsf/Content/Documents-for-Applicants-and-Assessment-Groups  [6] Medical Services Advisory Committee. 2021. ***ADAR Assessment Report Template***. [Online]. 19th May 2021 [Last accessed 7th July 2021]. Available from: http://www.msac.gov.au/internet/msac/publishing.nsf/Content/Documents-for-Applicants-and-Assessment-Groups  [7] Medical Services Advisory Committee. 2021. ***DCAR Assessment Report Template.*** [Online]. 19th May 2021 [Last accessed 7th July 2021]. Available from: http://www.msac.gov.au/internet/msac/publishing.nsf/Content/Documents-for-Applicants-and-Assessment-Groups  [8] Medical Services Advisory Committee. 2021. ***ADAR Commentary***. [Online]. 19th May 2021 [Last accessed 7th July 2021]. Available from: http://www.msac.gov.au/internet/msac/publishing.nsf/Content/Documents-for-Applicants-and-Assessment-Groups  [9] Medical Services Advisory Committee. (Page last updated 21st May 2021). ***MSAC Membership*** [Online]. Available from: http://www.msac.gov.au/internet/msac/publishing.nsf/Content/msac-membership [Last accessed 12th July 2021]. | MSAC is a nonstatutory committee established by the Australian Minister for Health in 1998, who’s role it is to provide recommendations to the Australian Health Ministry on the public funding of new or amended health services [2]. At MSAC HTA reports are termed ‘assessment reports’ and can be performed both by either the applicant or an external HTA group contracted on their behalf (Applicant–led assessment reports, ADARs) [6], or alternatively MSAC can contract an external HTA group directly (Department–led assessment reports, DCARs) [7]. The decision on which path to follow is informed by the applicant’s ability to undertake and engage in the HTA process [2, p.42]. External HTA groups also perform critical summaries of ADARs for consideration by MSAC [8]. |
| **National Institute for Health and Care Excellence Diagnostics Assessment Programme (NICE DAP, England and Wales)** | Medical tests, other than population screening tests | **Methods guide**^6^ specific to tests (other than population screening).  Other sources:  [1] Organisation website: <https://www.nice.org.uk/> [last accessed 18/06/21]  [2] ***Medical technologies evaluation programme methods guide.*** NICE; August 2017. Downloaded 11^th^ June 2020.  [3] ***Interim Addendum to the Diagnostics Assessment Programme Manual*.** NICE webpage, available from <https://www.nice.org.uk/Media/Default/About/what-we-do/NICE-guidance/NICE-diagnostics-guidance/Diagnostics-interim-addendum-access-proposals.pdf> [last accessed 5^th^ July 2021]  [4] **Interim addendum to replace existing section 9, Guidance reviews, in DAP programme manual.** NICE webpage, available from <https://www.nice.org.uk/Media/Default/About/what-we-do/NICE-guidance/NICE-diagnostics-guidance/Diagnostics-interim-addendum-guidance-reviews.pdf> [last accessed 5^th^ July 2021] | The national HTA body for England and Wales, NICE is accountable to but independent from the UK Government. NICE guidance technically only covers England but decisions about how NICE guidance applies in other countries in the UK (Wales, Scotland and Northern Ireland) is made by the respective devolved administrations. These other UK countries are often involved in and consulted on the development of NICE Guidance, and NICE has agreements to provide them with certain services and products.  HTA of tests is undertaken across three departments, though new technologies are primarily evaluated within the Medical Technology Evaluation Programme (MTEP) and Diagnostics Assessment Programme (DAP). MTEP receive proposals for diagnostic topics, and assess topics considered (by a topic briefing) to provide “*similar health outcomes at less cost, or improved health outcomes at the same cost as current NHS practice”*^6^ [2, pg. 11]. Complex tests, for example where recommendations can only be made on the basis of cost-effectiveness analysis, where HTA is of multiple technologies or patient indications, or tests likely to be more costly, are considered within the DAP [2, pg. 8 and pg. 11].  HTA within NICE is undertaken by independently commissioned academic groups (External Assessment Groups). |
| **Swedish Agency for Health Technology Assessment and Assessment of Social Services (SBU, Sweden)** | All healthcare interventions, including all medical tests. | **Methods guide**^7^ for all interventions, with test–specific chapters.  Other sources:  [1] ***SBU Method Book (google translation into English), October 2020 [downloaded 10th February 2021].*** *https://www.sbu.se/en/method/ [last accessed 15th April 2022].*  [2] ***Heintz E, Lintamo L, Hultcrantz M, Jacobson S, Levi R, Munthe C, et al. Framework for Systematic Identification of Ethical Aspects of Healthcare Technologies: The SBU Approach. Int J Technol Assess Health Care 2015;31(3):124–130.***  [3] ***Organisation website (English version): https://www.sbu.se/en [last accessed 10th March 2021]*** [8] ***The HTA network Sweden.*** Available from <https://www.sbu.se/en/collaboration/the-hta-network-sweden/> [last accessed 11^th^ August 2021]. | Health technology assessment in Sweden is led by its national body, the SBU. SBU together with SBU host a national HTA network where total 25 organisations participates (including the 21 regional HTA centres ) [3]. |
| **National Health Care Institute (Zorginstituut Nederland) (ZIN, Netherlands)** | All healthcare interventions, including all medical tests. | **Methods guide**^8^ specific to medical tests.  Other sources:  [1] ***Medical tests (assessment of established medical science and medical practice)***. Approved by CVZ on 20th January 2011. Publication number 293. [downloaded 23^rd^ March 2021]  [2] **Assessment of ‘established medical science and medical practice’.** Zorginstituut Nederland. Beoordeling stand van de wetenschap en praktijk. Diemen, 2015. [downloaded 22^nd^ March 2021].  [3] ***Assessment of ‘established medical science and medical practice’: a technical modification.*** Final version, 23^rd^ April 2019. Series number 2019003598. [downloaded 23^rd^ March 2021].  [4] ***Guideline for economic evaluations in healthcare.*** Final version, 16^th^ June 2016. Series number 2016077622. [downloaded 22^nd^ March 2021].  [5] ***Tasks of the National Health Care Institute.*** Zorginstituut Nederland’s web page, available from: <https://english.zorginstituutnederland.nl/about-us/tasks-of-the-national-health-care-institute> [last accessed 6^th^ May 2021].  [6] ***Working method for the Zinnige Zorg*** (Appropriate Care Programme), National Health Care Institute, August 2018. [downloaded 23^rd^ March 2021].  [7] ***Appropriate Care***. Zorginstituut Nederland’s web page, available from: <https://english.zorginstituutnederland.nl/zinnige-zorg> [last accessed 6^th^ May 2021]. | In the Netherlands, HTA is carried out by the Dutch National Health Care Institute (Zorginstituut) which is “an advisory and implementing organisation for two statutory health insurance schemes: the Health Insurance Act (Zorgverzekeringswet, Zvw) and the Long­Term Care Act (Wet Langdurige Zorg, Wlz)”[5]. The Institute “assesses whether diagnostics and (therapeutic) interventions are being deployed in a patient-oriented, effective and cost-effective manner.” Its guiding principle is that “Every citizen must be able to count on receiving good health care. No more and no less than is necessary, while also avoiding unnecessary costs.” [6, p.2]. General basic health insurance only includes care (including medical tests) that is considered effective according to the ‘established medical science and medical practice’ [1]. |

**Main sources:**

1. Methods Guide for Medical Test Reviews. AHRQ Publication No. 12-Ec017 . Rockville, Md: Agency for Healthcare Research and Quality; June 2012. Downloaded 20th July 2020.

2. Guidelines for Authors of CADTH Health Technology Assessment Reports. Revised May 2003. Ottawa: CADTH. [Downloaded 23rd September 2020].

3. Guidelines for the Economic Evaluation of Health Technologies: Canada. 4th Ed. Appendix — Specific Guidance for Treatments with Companion Diagnostics. Ottawa: Cadth; 2019 Sep. [Downloaded 24th July 2020].

4. General Methods (Version 6.0, 5th Nov 2020). Informal Translation of Final Draft of Allgemeine Methoden. (Available From: Https://Www.Iqwig.De/En/About-Us/Methods/Methods-Paper/). [Pdf Downloaded 12th December 2020].

5. Guidelines for Preparing Assessments for the Medical Services Advisory Committee. May 2021. Canberra: MSAC.

6. Diagnostics Assessment Programme manual. NICE; December 2011. Downloaded 28th April 2020.

7. SBU Method Book (Google Translation into English), October 2020 [Downloaded 10th February 2021].

8. Zorginstituut Nederland. Medical tests (assessment of established medical science and medical practice). Report no 293. Zorginstituut Nederland: Diemen, 2011. Available from: https://english.zorginstituutnederland.nl/publications/reports/2011/01/20/medical-tests-assessment-of-established-medical-science-and-medical-practice Last accessed 15th April 2022.
