# Supplementary material for: Health technology assessment of diagnostic tests: a state of the art review of methods guidance from international organizations
Source: Int J Technol Assess Health Care. 2023 Feb 21;39(1):e14. doi: 10.1017/S0266462323000065 (PMC7614237; doi:10.1017/S0266462323000065)
Supplement: Supplementary file 1 [file S0266462323000065sup001.zip › S0266462323000065sup005.docx]

**Supplementary Table 3: Special features in key organisations (N=7)**

| **HTA organisation** | **Special features noted in data abstraction for key organisations** |
| --- | --- |
| AHRQ | - Use of the AHRQ ‘analytic framework’ as a lynch pin of reviews. The framework is first developed during topic development (scoping), and is used to provide clarity, consistency and depth to the whole systematic review process. The framework is used to set out the ‘chain’ of outcomes that lead from the test itself to downstream health outcomes. - “HTA” by Evidence Practice Centers are conceived as systematic reviews of the evidence on tests possibly augmented by modelling. The system for making policy decisions/guidance does not sit within the remit of AHRQ, but with the US Centers for Medicare and Medicaid Services who make coverage decisions using AHRQ HTA reports. - Special attention given to the particular needs of genetic tests [1, Chapter 11] and prognostic tests [1, Chapter 12]. |
| CADTH | - Appendix dedicated to health economic evaluation of codependent tests. |
| IQWiG | - Strong preference for evidence based on RCTs even for tests, and that an absence of such evidence should result in a recommendation that it is not possible to make reliable recommendations. |
| MSAC | - Rigorously developed process framework [3.] that incorporates tailoring for HTAs of tests. - The health–related claims of testing underpin all elements of the HTA approach, and a structure is provided to assemble the claim in a standardised manner, the ‘assessment framework’. - The review of clinical evidence and cost–effectiveness analysis are explicitly linked by the claims of the test. - Specific and extended information on what is meant by a linked evidence approach [2. TG 11–13]. - Separating non-inferiority and superiority of accuracy, with some detailed guidance, e.g. Figure 9 [2. p.83] and Appendix 5 [2. p.267]. - Specific considerations of other test types throughout the Guidance, including screening, monitoring, prognosis, predictive, genetic and artificial intelligence/machine learning tests. - An additional special chapter is included for particular types of tests, discussing the following key considerations:   - “Screening can encompass broad or narrow populations. Considerations relevant to the assessment of universal screening programs, targeted screening, predisposition testing and cascade testing will differ [2. TG 15.1].   - Tests used to determine prognosis may have value relating to improved health outcomes if they inform change in management, or only have benefits that fall within the concept of value of knowing. An assessment of a prognostic test reports how well a test can differentiate future health events compared with current prognostic (often clinical) tests, as well as how this will impact clinical management [2. TG 15.2].   - Predictive tests are used to determine how well a patient would respond to a treatment. Typically, these tests identify a biomarker that inform the eligibility for a treatment and is most commonly applied in a codependent context. A critical concept within predictive testing is the clinical utility standard, and an assessment includes a robust comparison of the proposed test against the clinical utility standard [2. TG 15.3].   - The assessment of a test used for monitoring is similar to the assessment of tests for other purposes, using either a direct or linked evidence framework. However, additional aspects of the test, such as its responsiveness to changes in response to an intervention (signal to noise ratio), the detectability of long-term change, and the ease of use and interpretation of the test, should be discussed [2. TG 15.4].   - An assessment of a multifactorial algorithm involves the presentation of the biological plausibility, the characteristics of the training and validation sets (for establishing applicability), and additional results to ensure that algorithms (particularly dynamic algorithms) are not subject to bias when used in the Australian population [2. TG 15.5].   - A codependent submission is required when the Minister for Health requires advice from 2 different expert advisory committees because listing of the co-dependent technologies involve 2 separate reimbursement schemes [2. TG 15.6].” - Clarification of the specific circumstances in which expert opinion may be useful [2. Appendix 9]. - Specific consideration of impacts that are not health–related: Equity [2. TG 29], value of knowing [2. TG 28], presence of effective alternatives [2. TG 29.7], organisational issues including efficiency of health delivery, ethical concerns, social aspects and legal concerns [2. TG 29]. Environmental aspects are also mentioned (e.g. reduction in emissions) [2. TG 29.6]. |
| NICE DAP | - Strong attention to process. - Prominent involvement of patients. - Extensive scoping phase involving stakeholders and care pathway research. - Emphasis on the use of modelling to assess whether the test is clinically and cost-effective – this is the means by which “evidence linkage” is achieved by NICE. - Final decision on recommendation relies heavily on the estimated cost-effectiveness (consistent with other guidance streams in NICE). - Use of expert elicitation. |
| SBU | - Preference for the term ‘diagnostic reliability’, rather than ‘diagnostic accuracy’. - Use of GRADE approach, modified by SBU, to weight different types of evidence on tests. - Systematic consideration of ethical considerations for all health technologies, including tests. |
| ZIN | - A framework for comparative analysis when no direct evidence is available. - A framework for assessment of a technical modification of an intervention whose effectiveness has been established or is not open to discussion. |

TG = technical guidance.

[1.] Methods Guide for Medical Test Reviews. AHRQ Publication No. 12-Ec017 . Rockville, Md: Agency for Healthcare Research and Quality; June 2012. Downloaded 20th July 2020.

[2.] Medical Services Advisory Committee. 2021. Guidelines for preparing assessments for the Medical Services Advisory Committee. [Online]. Version 1.0. [Last accessed 16th June 2021]. Available from: http://www.msac.gov.au/internet/msac/publishing.nsf/Content/Documents-for-Applicants-and-Assessment-Groups

[3.] Medical Services Advisory Committee (MSAC) Reform Implementation. 2016. Process Framework. [Online]. Version 1.0. [Last accessed 20th July 2021]. Available from: http://www.msac.gov.au/internet/msac/publishing.nsf/Content/msac-process-framework
